# Face Patch Resting State Networks Link Face Processing to Social Cognition

**DOI:** 10.1371/journal.pbio.1002245

**Published:** 2015-09-08

**Authors:** Caspar M. Schwiedrzik, Wilbert Zarco, Stefan Everling, Winrich A. Freiwald

**Affiliations:** 1 Laboratory of Neural Systems, The Rockefeller University, New York, New York, United States of America; 2 Robarts Research Institute, University of Western Ontario, London, Ontario, Canada; University of Oxford, UNITED KINGDOM

## Abstract

Faces transmit a wealth of social information. How this information is exchanged between face-processing centers and brain areas supporting social cognition remains largely unclear. Here we identify these routes using resting state functional magnetic resonance imaging in macaque monkeys. We find that face areas functionally connect to specific regions within frontal, temporal, and parietal cortices, as well as subcortical structures supporting emotive, mnemonic, and cognitive functions. This establishes the existence of an extended face-recognition system in the macaque. Furthermore, the face patch resting state networks and the default mode network in monkeys show a pattern of overlap akin to that between the social brain and the default mode network in humans: this overlap specifically includes the posterior superior temporal sulcus, medial parietal, and dorsomedial prefrontal cortex, areas supporting high-level social cognition in humans. Together, these results reveal the embedding of face areas into larger brain networks and suggest that the resting state networks of the face patch system offer a new, easily accessible venue into the functional organization of the social brain and into the evolution of possibly uniquely human social skills.

## Abstract 2

An analysis of the functional connectivity of regions of the monkey brain involved in face recognition suggests substrates for the cognitive, mnemonic, emotive, and motoric impact of faces, revealing striking similarities to the human brain, and implying a deep evolutionary heritage of even the most high-level sociocognitive functions.

## Author Summary

Primates have evolved to transmit social information through their faces. Where and how the brain processes facial information received by the eyes we now understand quite well. Yet we do not know how this information is made available to other brain areas so that a face can evoke an emotion, activate the memory of a person, or draw attention. Here, to identify brain regions interacting with face areas, we performed whole-brain imaging in macaque monkeys, whose face-processing system we know best. We find that the core face-processing areas are connected to several other brain areas supporting socially, emotionally, and cognitively relevant functions. Together, they form an extended face-processing network, similar to what has been proposed for humans. This extended face-processing network intersects with a second large-scale network, the so-called “default mode network”, in a pattern stunningly similar to that in the human brain. This intersection identifies selectively those brain regions that implement the most high-level forms of social cognition, such as understanding others’ thoughts and feelings. Thus, the results of this novel approach to understanding the functional organization of the social brain point to a deep evolutionary heritage of human abilities for social cognition.

## Introduction

Primates are highly social animals who cope with the challenges posed by life in complex social groups through sophisticated mechanisms for the recognition, evaluation, and generation of social signals. To understand the neural circuits mediating primate social behavior, here we take a novel, bottom-up approach utilizing a particularly well-defined sensory circuit as our starting point: the neural machinery that processes faces. Faces transmit rich information about socially relevant dimensions such as personal identity, emotional expressions, and gaze direction [1]. To extract this multidimensional facial information, primates have evolved specialized brain areas [2], which are tightly and specifically interconnected [3], to form a face-processing system. Yet how this face-processing system is embedded, and thus how it makes face information available to other systems supporting social and cognitive functions, is largely unknown.

The connections between face processing and cognition are important to understand because behavioral and developmental studies show that faces occupy a special status among other, socially less relevant objects. Faces selectively draw spatial attention [4] and attract saccades much faster than other objects do [5], indicating privileged routing of facial information into attentional and eye movement control systems. Faces also drive specific mnemonic, emotional, and communicative responses [6], again suggesting specialized circuitry linking face areas to recipient, non-face-selective areas elsewhere in the brain.

To reveal with which parts of the brain the face-processing system can exchange information, we used resting state functional magnetic resonance imaging (rsfMRI) seeded in functionally defined face areas of the macaque monkey, the main animal model for face processing. rsfMRI noninvasively measures functional connectivity (FC) between brain areas based on spontaneous low frequency activity correlations, at high spatial resolution, and with full brain coverage [7]. We focus on FC because although FC generally shows good agreement with anatomical connectivity [8], the set of potential functional connections between brain areas is far greater than that of direct structural links, as it is not constrained to monosynaptic connections but also includes dynamic, polysynaptic connectivity [9]. A previous study of face patch connectivity using electrical microstimulation, a method that reveals primarily monosynaptic connectivity, found face patches to form a structurally closed system with few output connections [3], thus begging the question how the face patch system interacts with other systems. rsfMRI provides this type of complementary information and—due to its wide use in basic and clinical research in humans—confers the additional advantage of being readily comparable between species [10], thus enabling insight into the evolution of face-recognition systems [2,11,12].

Face area FC maps are not only essential for understanding the neural mechanisms of face recognition, they also provide a unique inroad into understanding high-level social cognition and its evolutionary heritage. This is because face areas are thought to constitute a major input into the so-called “social brain”, a set of brain areas devoted to the processing of social interactions [13]. In fact, almost 60% of the variance in our attitudes towards others can be explained by facial information alone [14]. In humans, one of the social brain’s core regions is the temporoparietal junction (TPJ). The TPJ is thought to be critical for high-level social cognition, in particular theory of mind (TOM) [15], the capacity to attribute mental states to ourselves and others. Apes and monkeys display basic forms of TOM such as understanding what others see or know [16,17]. However, the very existence of a TPJ homolog in monkeys has been debated since the days of Brodmann, in part because the high-level functions that are supported by human TPJ, such as understanding others’ false beliefs, may not be present in the macaque [15,18]. This uncertainty is in large part due to the difficulties in studying macaque social cognition in a controlled experimental setup [19,20]. Our approach sidesteps this issue and enables us to assess whether the kind of processing architecture enabling social cognition in humans already exists in the macaque: if this architecture was entirely absent, this would indicate that certain aspects of social cognition are indeed uniquely human. Conversely, if we could uncover similar brain networks in the macaque, this would suggest that at least a minimal scaffolding for high-level social cognition is already present in a primate whose evolutionary lineage split from ours some 25 million years ago [21].

To achieve this goal, we make use of the fact that the human social brain overlaps with another large scale network, the so-called default mode network (DMN) [22–24]. The DMN, readily identifiable with rsfMRI in both humans and monkeys [25,26], comprises a set of interconnected areas more active during rest than task performance and is thus thought to generate the brain’s default activity [27]. Importantly, the overlap between the human DMN and the social brain includes high-level social cognition areas like TPJ, medial posterior parietal cortex (PPC), and dorsomedial prefrontal cortex (dmPFC) [23,28]. We thus assessed, using the face patch resting state networks as a proxy for the social brain, whether and where a similar overlap exists in the macaque brain. This approach allows us to identify candidate homologs of human high-level social cognition brain areas.

## Results

To determine face patch resting state networks (FPRSNs), we first identified face patches in six awake macaque monkeys using standard face localizers. We presented pictures of faces, bodies, and other object categories and contrasted activation during face presentations with activations during the presentation of nonface stimuli to reveal face areas. We identified one orbitofrontal (prefrontal orbital, PO) [29] and five temporal (middle lateral, ML; middle fundus, MF; anterior lateral, AL; anterior fundus, AF; anterior medial, AM) [2] face patches in all six animals. For subsequent analyses, whenever possible, bilateral homolog pairs were joined into one region of interest (ROI). Subsequently, the same monkeys underwent scans for rsfMRI during light isoflurane anesthesia. Aligning face localizer with rsfMRI scans allowed us to extract the time courses of spontaneous activity from each of the face patches and from regions outside of the face patch system.

**Fig 1:**
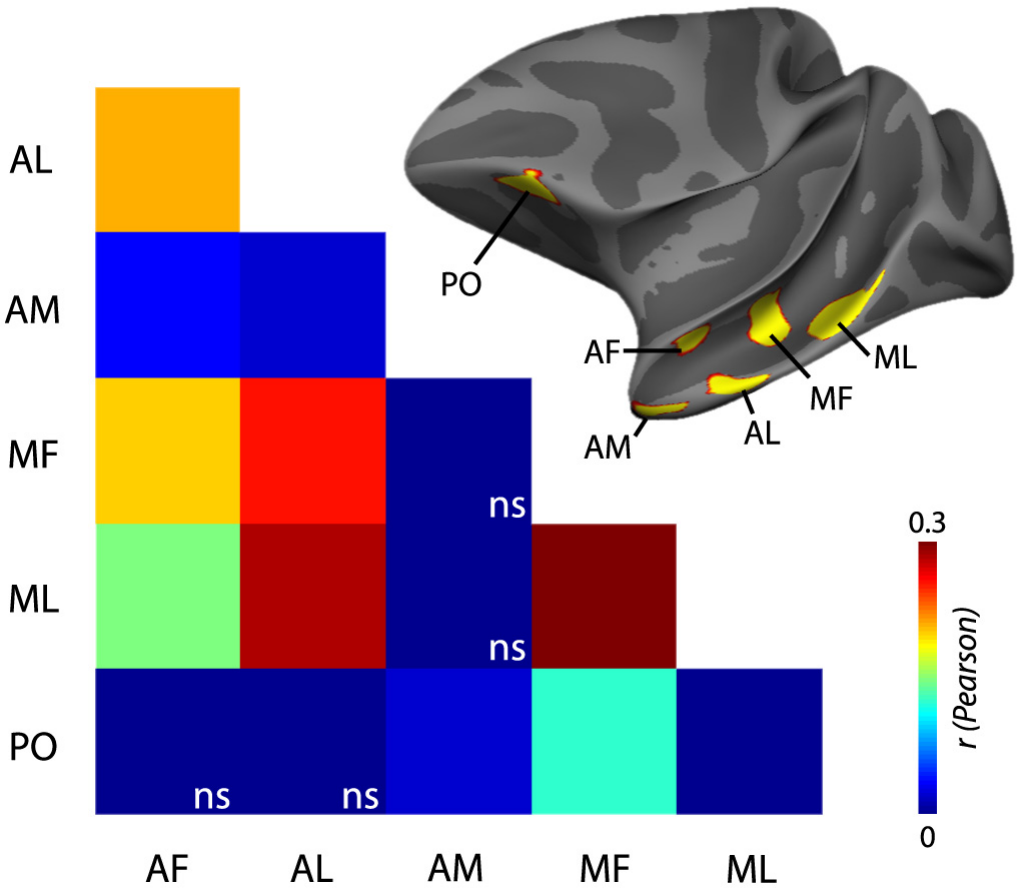
FC within the face patch system. Most face patches were functionally connected to each other, with the exception of the frontal face patch PO, which was only connected to AM, MF, and ML and AM, which was only connected to AF, AL, and PO (corrected for multiple comparisons using FDR at *q* = 0.05). The inset shows the location of the face patches of an example subject on the average surface of the left hemispheres of the rhesus macaques used in this study. Nonsignificant correlations (ns) are set to 0.

As a first step towards characterizing FPRSNs, we determined connections within the face patch system. Our goals were to (i) reveal the hitherto unknown FC between temporal and orbitofrontal face patches, (ii) determine whether the established organizational principles of the temporal lobe face patch system, i.e., hierarchical and parallel organization, can be recovered from rsfMRI, and (iii) validate the intra-face patch connectivity pattern our noninvasive methodology reveals with data from an invasive approach, i.e., electrical microstimulation. To this end, we performed ROI-to-ROI correlation analyses after regressing out motion, heartbeat, and breathing artifacts from the data. We found FC between all face patches, with average Pearson correlation coefficients ranging between 0.34 for ML-MF and 0.02 for ML-AM (Fig 1, Wilcoxon signed rank tests, one-sided, corrected for multiple comparisons using the False Discovery Rate (FDR) at *q* = 0.05). The frontal patch PO, whose connectivity pattern was previously unknown, showed significant FC with the temporal patches MF, ML, and AM. AM, the face patch residing at the top of the temporal face-processing hierarchy [30], showed significant FC with PO, a likely output structure for AM, and with AF and AL, two input areas to AM located at the preceding level of the processing hierarchy, but not ML and MF, which are one level further removed. Thus, rsfMRI FC patterns recover the first organizational principle of the face-processing system, i.e., its hierarchical organization along the posterior–anterior axis of the superior temporal sulcus (STS). The second main organizational principle of the face patch system is parallelism: two processing streams reside in different cytoarchitectonic subdivisions of the STS, one in the fundus and the other in the lower bank of the STS. Hence, we tested whether the strength of FC followed known anatomical patterns of connectivity [31], i.e., whether face patches residing either in the fundus or on the lip of the STS (MF-AF and ML-AL) are more strongly correlated with each other, or whether their connectivity was equally strong across the fundus and the lip of the STS (AF-ML and AL-MF). Indeed, we found stronger FC within cytoarchitectonic subdivisions than across (median_HL_ difference CI_95_ = [0.004 0.146]; Wilcoxon signed rank test, *p* = 0.03, two-sided), but this effect was mainly driven by the differential connectivity of ML. Anatomy also predicts a small but systematic bias for stronger interhemispheric connectivity between homolog than nonhomolog brain areas [32], and interhemispheric FC was in fact slightly higher between homolog face patches than between nonhomolog face patches (median_HL_ difference CI_95_ = [0.0037 0.2326]; Wilcoxon signed rank test, *p* < 0.02, one-sided). Finally, we compared the pattern of FC between the face patches from rsfMRI to previous results obtained with electrical microstimulation [3], which had revealed a highly specific set of connections between the STS face patches. As these specialized anatomical links provide the major scaffold for the functionally defined face processing network, we expected that rsfMRI should reveal a similar (although not identical) pattern of FC. A Spearman rank correlation between connection strengths indeed showed a high level of agreement between the two methods (*r* = 0.6314, *p* = 0.01; S1 Fig). The same results were obtained using the robust correlation method Shepherd’s Pi (*r* = 0.6352, *p* = 0.02) [33]. This substantiates the noninvasive rsfMRI approach with results from a method known for its specificity in identifying direct neuronal connections [34].

**Fig 2:**
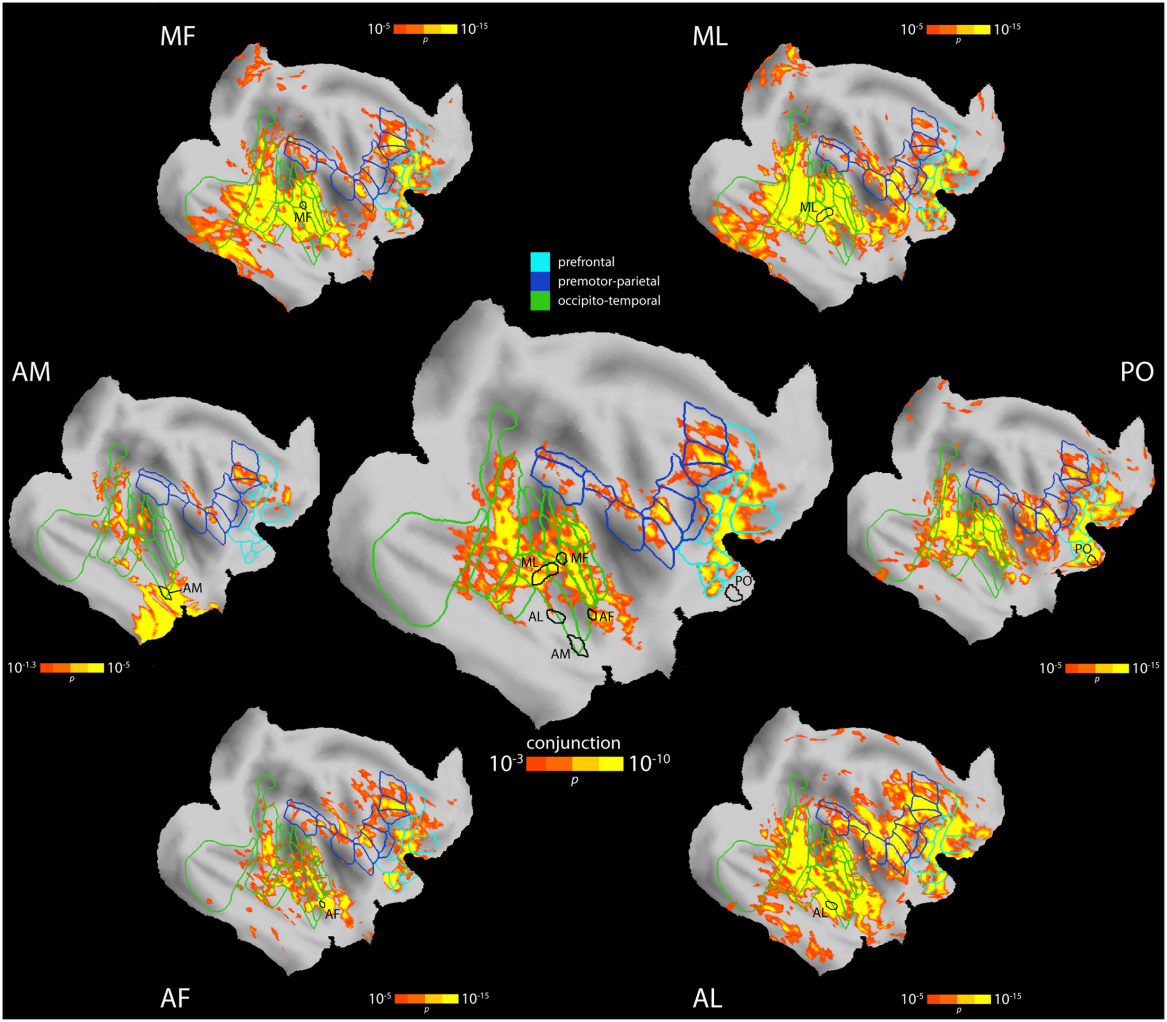
Cortical FC of the face patches. The central panel shows the results of a conjunction analysis of the maps from AF, AL, MF, ML, and PO (corrected for multiple comparisons using cluster size thresholding at *p* < 0.05) on an inflated and flattened right hemisphere in F99 space. Highlighted are three broad networks of FC: areas in the prefrontal cortex (light blue), a premotor-parietal network (blue), and an occipitotemporal network including ventral stream areas (areal boundaries from Lewis & van Essen [35]). The surrounding panels show connectivity maps of the individual face patches (corrected for multiple comparisons using cluster size thresholding at *p* < 0.05), along with the areal boundaries of the conjunction analysis (light blue, blue, green). Representative locations of the face patches are outlined in black. See Table 1 for a list of area names, S4 Fig for complete areal labeling of face patch connectivity on the conjunction map based on Lewis & van Essen [35], S5 Fig for areal labels of the premotor-parietal network based on Paxinos et al. [38], S6 Fig for a quantification of how many face patches connected with a given vertex, S7 Fig for a conjunction analysis restricted to the temporal face patches (AF, AL, MF, ML), and S8 Fig for results of a corresponding conjunction analysis in volume space. Data shown here are publicly available at the Dryad Digital Repository [39].

The second main goal of this study was to determine the embedding of the face patches into the rest of the brain. We first focused on connections with other cortical areas. To this end, we aligned and brought all functional data into a common surface space, preserving high specificity for cortical grey matter and anatomical landmarks despite slight smoothing (1.25 mm kernel). We then performed a fixed effects (FFX) General Linear Model (GLM) group analysis for each of the face patches, respectively. After correction for multiple comparisons, these analyses revealed a highly convergent set of connected areas for AL, AF, MF, ML, and PO (and the posterior lateral face patch, PL, which was identified only in a subset of animals, S2 Fig), as well as connections that were unique to individual face patches (Fig 2, S3–S9 Figs). The FC pattern common to all face patches can be summarized by a conjunction analysis using the minimum statistic from each of the five maps (Fig 2, center). Both temporal and orbitofrontal face patches were connected to the (i) lateral prefrontal cortex (Fig 2, light blue), including areas 12 and 46, where face-selective neurons have been located [36]; (ii) regions of premotor cortex (Fig 2, blue), including areas F2, F4, F5, and F7, involved in the visual guidance of movements [37]; (iii) inferior parietal areas, including areas 7a and 7b (Fig 2, blue); (iv) areas of the temporal lobe, including the lower bank, fundus, and upper bank of the STS and parts of area TE (Fig 2, green); and (v) early visual cortex, especially areas V3 and V4 (Fig 2, green). This pattern of results was highly consistent across hemispheres (S3 Fig). Within this common pattern of connectivity shared across face patches, we also found significant variation across face patches (S9 Fig): (i) connectivity to the insula was more prominent for AL, ML, and PO than for MF and AF, (ii) AL and AF connectivity extended more posteriorly on the dorsolateral surface towards the central sulcus than any of the other face patches, and (iii) only AM showed connectivity to medial temporal lobe structures (entorhinal cortex, perirhinal cortical areas 35 and 36).

**Table 1:** Areas right hemisphere. Area names are from Lewis & van Essen [35].

Prefrontal	Premotor-parietal	Occipito-temporal
11l	4C	FST
12	6DR	IPa
13l	6Ds	MSTda
13m	6M	MSTm
45	6Val	MT
46p	6Vam	TAa
46v	6Vb	TE1-3
8Ac	7a	TEa-m
8As	7b	TPOc
9	LIPd	TPOi
	PrCO	TPOr
	S2	Tpt
		Ts
		V2v
		V3
		V4
		V4ta
		V4tp
		VOT
		VP

**Fig 3:**
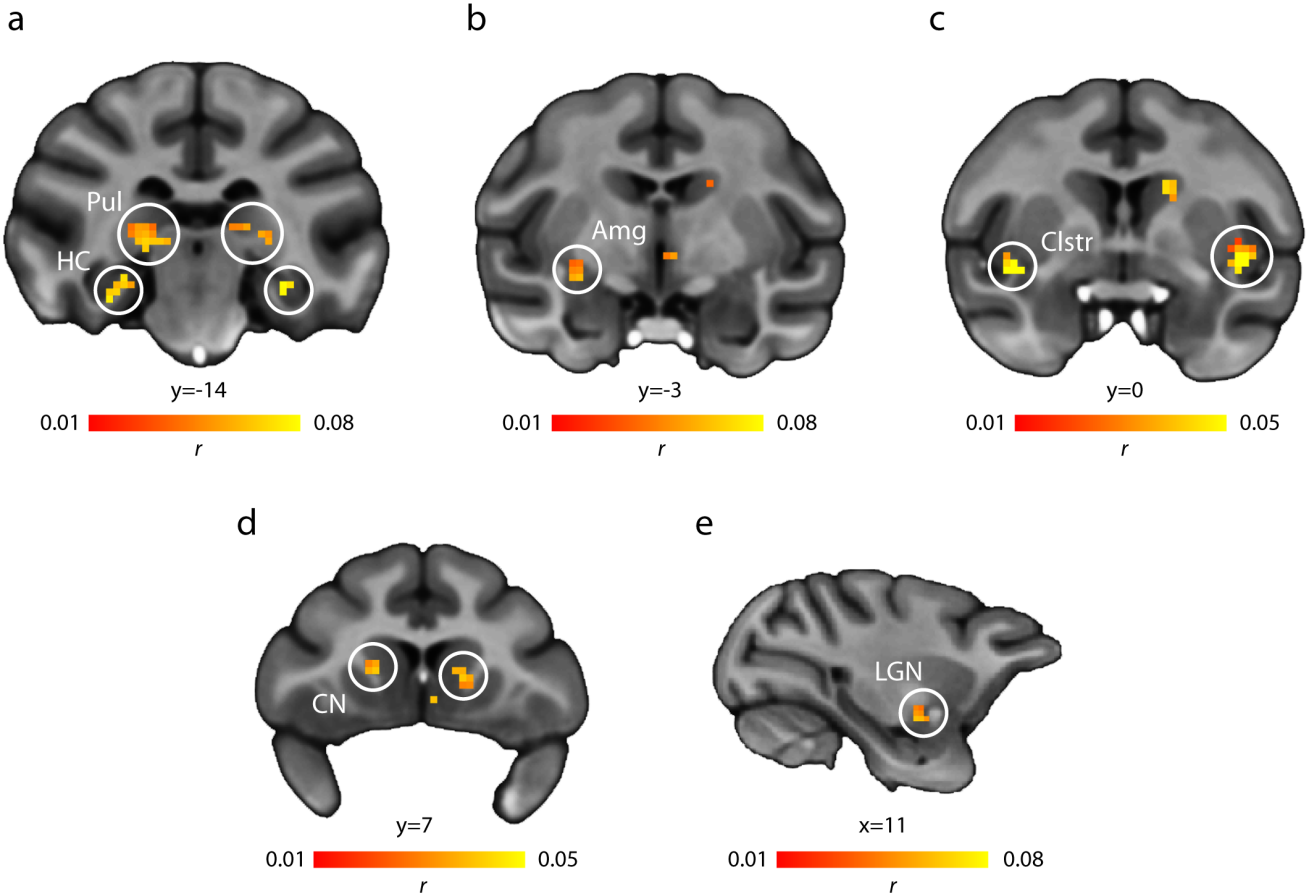
Subcortical FC of the face patches. A conjunction analysis of the rsfMRI maps of face patches AF, AL, MF, ML, and PO revealed FC with several subcortical areas. (a) Pulvinar and hippocampal formation (FDR corrected at *q* = 0.025); (b) amygdala (FDR corrected at *q* = 0.005); (c) claustrum (FDR corrected at *q* = 0.025); (d) caudate nucleus (FDR corrected at *q* = 0.025); (e) lateral geniculate nucleus (FDR corrected at *q* = 0.005). Results are overlaid on coronal (a–d) and sagittal (e) slices of the MNI-Paxinos template brain, in radiological convention (left is right). Coordinates are relative to the center of the anterior commissure. Cortical results are masked for display purposes. Note different scaling of correlation coefficients in (c) and (d).

Face-selective neurons have been found in several subcortical structures such as the pulvinar [40] and the amygdala [41], which are not accessible to surface-based analyses. To determine FC of the cortical face patch system with subcortical structures, we performed a volume-based whole-brain analysis. After smoothing (2 mm Gaussian kernel), a FFX GLM revealed subcortical FC of the face patch system with a subregion of the claustrum, the amygdala, and the pulvinar, as had previously been shown using microstimulation [3], and additionally with the geniculate nucleus of the thalamus, the caudate nucleus, and the hippocampal formation (Fig 3).

**Fig 4:**
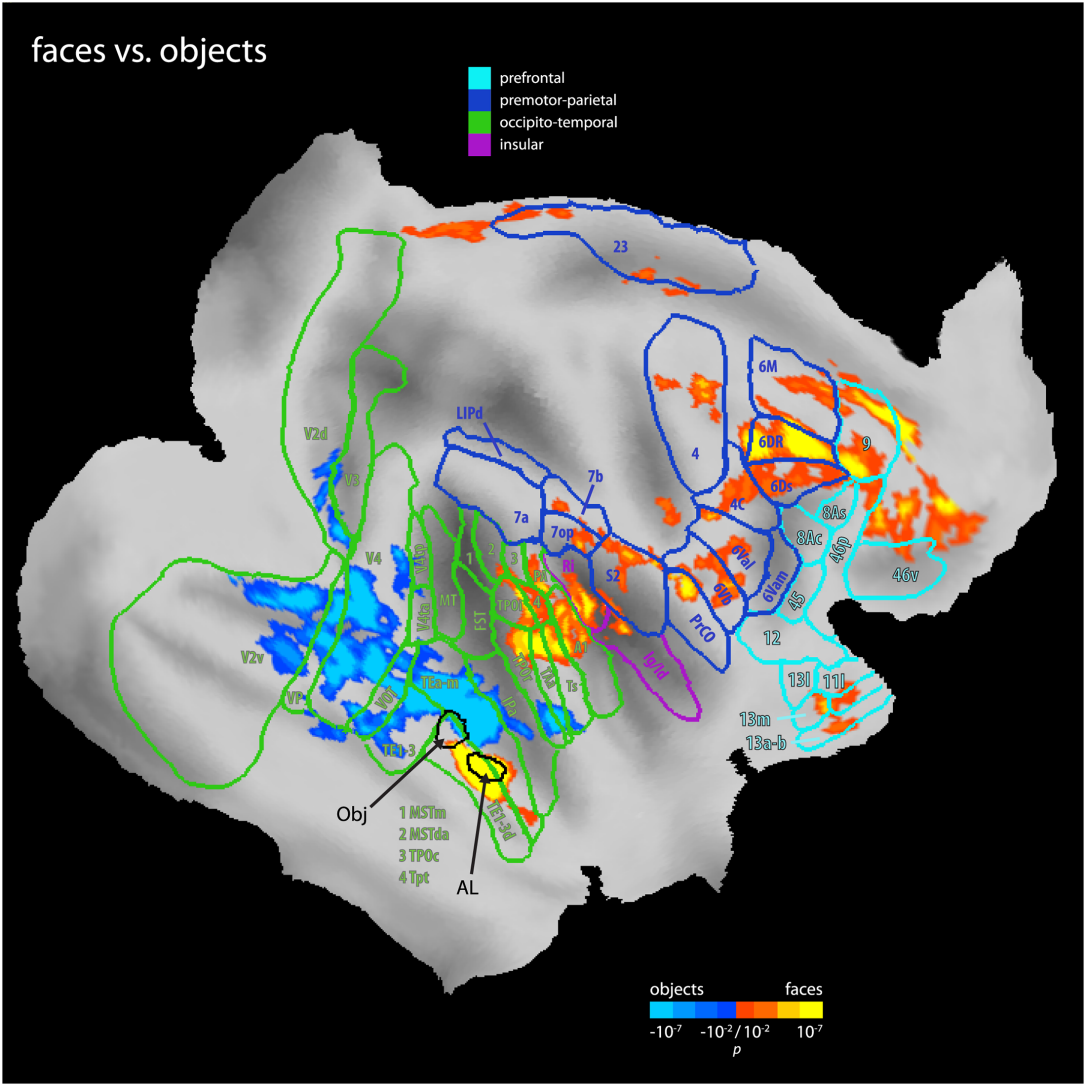
Differential FC of face versus object patches. Results of the contrast of face patch AL against an object patch connectivity (corrected for multiple comparisons using cluster size thresholding at *p* < 0.05) on an inflated and flattened right hemisphere in F99 space. The two seed regions were neighboring each other in the anterior lip of the STS (representative locations marked with arrows and black outlines, average distance 4.1 mm). Warm colors show areas in which face patch connectivity was stronger than object patch selectivity, while cold colors show the reverse. As can be seen, the face patch AL showed a connectivity pattern distinct to that of the object patch, despite their close proximity. AL was more connected with the upper bank of the STS (including area TPO), the insula, lateral, and medial parietal cortex (including area 7 and 23, respectively), lateral and medial prefrontal cortex (including areas 6Val, 6Vb and 6M, 9, respectively), and orbitofrontal cortex (area 13). In contrast, the object patch was more connected with the inferotemporal cortex (including area TE) as well as the ventral occipital cortex (including area VOT). Highlighted are four networks of FC: areas in prefrontal cortex (light blue), a premotor-parietal network (blue), occipitotemporal cortex (green), and the insula (purple). Areal boundaries are from Lewis & van Essen [35] and include all areas from the conjunction of face patches analysis (Fig 2) for comparison.

It has been suggested that visual categories represented in spatially disjunct parts of ventral visual cortex are associated with unique patterns of connectivity [42]. We thus tested which of the functional connections we observed were specific to the face patches. To this end, we isolated a patch in the anterior lip of the STS that responded to manmade objects during the localizer scans and then contrasted its FC to that of anatomically neighboring face patch AL (see Materials and Methods). AL showed stronger connectivity with the upper bank of the STS (including area TPO), the insula, lateral and medial parietal cortex (including area 7 and 23, respectively), lateral and medial prefrontal cortex (including areas F4, F5 and 6M, 9, respectively), as well as orbitofrontal cortex (area 13) (Fig 4). In contrast, the nearby object patch showed stronger connectivity with the inferotemporal cortex (including area TE) as well as the occipital cortex (including area VOT). Hence, faces and objects indeed display distinct patterns of FC.

Overall, the FC pattern of the face-patch system we identified includes areas like the lateral prefrontal cortex and the amygdala that contain face representations themselves, and others, like the premotor cortex, that likely do not. How strongly then does functional specialization for faces shape face patch FC? Specifically, we tested whether the strength of FC on the whole-brain level depended on the selectivity of the target voxels, i.e., whether face patches were more connected to other face-selective voxels than to object-selective voxels. First, as a measure of selectivity, we computed *d’* between faces and objects for each voxel from the localizer data. Because rsfMRI connectivity falls off with distance, we also calculated the Euclidean distance from the voxel with peak selectivity within each respective face patch to the remaining voxels within the same hemisphere. We then matched voxels outside the face patch under consideration for selectivity and distance, weighing both factors equally (see Materials and Methods). Finally, we compared FC with matched face and object-selective voxels across 12 hemispheres, and found that for each face patch (AF, AL, MF, ML, PO), connection strength was higher for face than object voxels (mean differences: AF 0.04, AL 0.03, MF 0.04, ML 0.02, PO 0.03, paired *t* tests, all *p* < 0.03, one-sided). This shows that whole-brain rsfMRI can recover functional specificity within connection patterns, similar to what has been shown for structural connectivity in humans [43].

**Fig 5:**
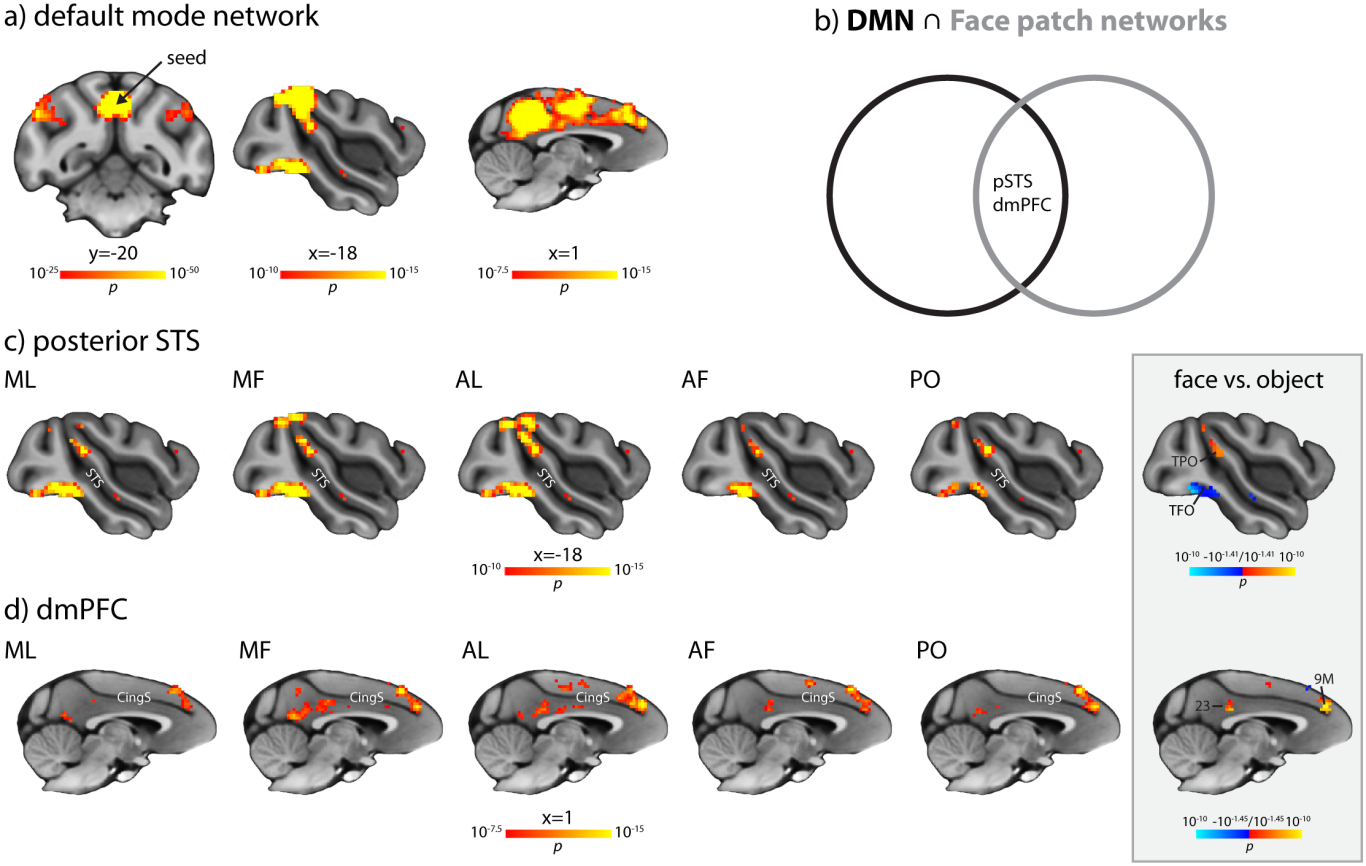
Overlap analysis of the DMN and FPRSNs. (a) The classical monkey DMN on coronal and sagittal slices. The arrow indicates the approximate location of the DMN seed. Note that the sagittal slices are the same as in c and d, respectively, at corresponding statistical thresholds. (b) Illustration of the overlap analysis. In humans, the intersection between the DMN and the social brain isolates areas involved in high-level social cognition, such as TPJ in the posterior STS, dmPFC, and medial PPC [22–24]. We tested, using the FPRSNs as a proxy for the social brain, whether and where a similar overlap exists in the macaque. (c) Voxels in area TPO in the dorsal bank of the posterior STS, located dorsally of FST, laterally to MST, and anterior and dorsal of MT, that show significant connectivity both with the PPC and with the respective face patch (AF, AL, MF, ML, and PO) at *p* < 10^−10^, uncorrected. The corresponding Jaccard Indices are 0.1358 for AF, 0.1635 for AL, 0.1786 for MF, 0.1366 for ML, and 0.1150 for PO. Also evident is overlap around the occipitotemporal sulcus, which is not part of the classical DMN. The grey inset shows that the strength of connectivity to voxels in which AL showed overlap with the DMN in the posterior STS was significantly higher for AL than for the object patch. In contrast, connectivity to the occipitotemporal sulcus was significantly higher for the object patch than for AL. (d) Voxels in dmPFC (areas 9M/10) and medial PPC (areas PGm/23) that show significant connectivity both with the PPC and with the respective face patch (AF, AL, MF, ML, and PO) at *p* < 10^−7.5^, uncorrected. The corresponding Jaccard Indices are 0.2006 for AF, 0.2268 for AL, 0.2530 for MF, 0.2062 for ML, and 0.1821 for PO. The grey inset shows that the strength of connectivity to voxels in which AL showed overlap with the DMN in the dmPFC (area 9M) and medial PPC (area 23) was significantly higher for AL than for the object patch. Both inset maps are corrected for multiple comparisons with a FDR at *q* = 0.05, accounting for the number of voxels that show significant overlap between AL and the DMN at the same statistical thresholds as shown in panels c and d, respectively. All results are overlaid on the MNI-Paxinos template brain, in radiological convention (left is right). Coordinates are relative to the center of the anterior commissure. Area labels are based on Paxinos et al. [38]. See S10 Fig for overlap between the DMN and AM connectivity, which replicates the main findings at a more lenient statistical threshold.

Can the neural systems that support the most advanced human sociocognitive skills be traced back to the macaque, a species with more limited sociocognitive abilities? In humans, social information processing networks, broadly defined, and the DMN overlap in the three cortical areas supporting the most high-level social-cognitive functions [22–24]. Should the two networks intersect in macaques as well, and should this intersection occur at anatomical locations corresponding to those in humans, this would support a scenario of deep evolutionary heritage of these sociocognitive abilities. We first determined the DMN according to its original definition [25], i.e., by seeding rsfMRI in a bilateral ROI placed in medial PPC (areas 31/PGm, see Materials and Methods). As in previous studies [25,26,44], we observed a network comprising PPC, medial prefrontal, and lateral temporoparietal cortex (Fig 5a). We then computed conjunction maps between the FPRSNs and the DMN. Fig 5c shows that there is significant overlap in area TPO in the posterior STS where the human TPJ resides. This result replicates for all face patches (including AM, S10 Fig). Furthermore, there is also overlap in areas 9M/10 in the dmPFC and, although less consistent in its precise location for each of the FPRSNs, areas PGm/23 in the medial PPC (Fig 5d), two further areas involved in high-level social cognition for which overlap with the DMN has been observed in humans [23]. Because the overlap critically depends on the statistical threshold at which it is evaluated, we calculated Jaccard indices, which can be interpreted as percent overlap between two networks, over a wide range of uncorrected thresholds and compared the empirically observed degree of overlap with distributions of Jaccard indices obtained from the overlap of the FPRSNs with randomly generated maps that had the same spatial and statistical properties as the DMN. After correction for multiple comparisons, we found significant overlap between the DMN and all FPRSNs, including that of AM, until thresholds were so conservative that the likelihood of overlap was minimal (S11 Fig). To assess whether the overlap with the DMN was specific to the FPRSNs, we also calculated the overlap between the object patch resting state network and the DMN. There was significantly more overlap between the FPRSN of AL and the DMN than between the object patch resting state network and the DMN at all thresholds (FDR-corrected, *q* = 0.01), and in fact, for most thresholds tested, there was no overlap between the object patch resting state network and the DMN at all (S12 Fig). Furthermore, voxels which showed overlap between the FPRSN of AL and the DMN in posterior STS, dmPFC, and medial PPC were more strongly connected with AL than with the nearby object patch, while the opposite was the case for a region around the occipitotemporal sulcus, an area in which FPRSN and DMN also overlapped but that is not considered part of the monkey DMN (Fig 5, grey inset). Together, these results show that the macaque FPRSNs and DMN prominently and specifically overlap in brain areas that support high-level social cognition in the human brain.

## Discussion

We find that the macaque face patches form a network linking highly face-selective regions in the temporal and orbitofrontal cortices. This face patch network is functionally embedded into a larger-scale, anatomically specific network of cortical and subcortical structures. This extended network significantly overlaps with the DMN, in particular in posterior STS, medial PPC, and dmPFC, which are involved in high-level social cognition in humans. The overlap is specific to the face patches, considering both the amount of overlap as well as the strength of FC in comparison to object resting state networks. Our results show the utility of a combined fMRI-rsfMRI approach in determining the embedding of functionally specific brain areas into larger-scale brain networks, and indicate that the face patch system may provide a unique inroad into understanding the complex organization of the social brain and a window into the evolution of primate social cognition.

### An Extended Face-Processing System in the Macaque Monkey

Human face recognition has been proposed to rely on a “core system” consisting of interconnected face-selective areas and an “extended system” that utilizes inputs from the core system for cognitive, emotive, and mnemonic functions [6]. Our whole-brain FC maps of the macaque face-processing system (Figs 2 & 3) show how the face patches are embedded into a larger-scale network that shares, as we will discuss below, many of the properties of the proposed human extended system. In addition, these maps show how the face patches are nested into the general flow of information along the visual ventral stream. The ventral stream is organized along a main posterior–anterior axis, and along a dorsal–ventral axis with extensive lateral connectivity [45]. This connectivity pattern can account for the coupling of face patches to occipital and temporal areas we observed and likely reflects the input–output relationships directly relevant for visual face-processing. A subcortical face-processing system has been proposed to exist, consisting of the superior colliculus, the pulvinar, and the amygdala [46]. FC of the face-processing system with pulvinar and amygdala, as we found here, is compatible with this proposal of a separate, nonclassical set of subcortical inputs into the cortical face-processing network.

The face patch system strongly interconnects with lateral prefrontal cortex, one of the main recipients of ventral stream output [45]. Since connectivity from ventral stream face patches appears to include, but does not appear to be confined to, face specializations within the lateral prefrontal cortex [29,36] and is only partially specific to the face patches, facial information is likely made available for both face-domain-specific processing like face-specific working memory [36] and for domain-general cognitive processes in prefrontal cortex-like categorization [47] or attentional control [48]. Further structures guiding spatial attention and eye movements are the supplementary eye field (SEF) in area F7, PPC, the pulvinar, the amygdala, and the caudate nucleus [49], all of which we found to be connected with the core face-processing system. These connections may aid in relaying information about the direction of attention of others extracted from a visual analysis of eyes and faces into the attentional system [50], and stronger connectivity of face patches than of nearby object patches with areas such as the SEF may underlie the behavioral advantages in directing saccades [5] and drawing spatial attention towards faces [4]. Thus, the core face-processing system interfaces with attention and executive control systems through multiple functional routes.

Extensive further connectivity of the face patch system to executive systems beyond those for oculomotor control was evidenced by face patch-specific rsfMRI connectivity to several parts of premotor cortex. This came as a surprise to us, since it was not predicted by classical anatomy. Current anatomical evidence for direct connections between the parts of the STS that contain the face patches is limited to area F7 [51,52]. Polysynaptic projections, however, from the STS to area F2 have recently been identified [53]. The latter may arise from relay through the ventrolateral prefrontal cortex [53] or through well-established connectivity between parietal areas 7b and S2, which provide input to areas F4 and F5 [37]. These premotor areas all contain visually responsive neurons and have been shown to be involved in the visual guidance of movements of face, eyes, and upper limbs; in particular, area F5 contains mirror neurons for socially relevant movements of the mouth such as lip smacks [54]. Thus, connectivity between face and premotor areas may be stronger than previously thought and may support social communicative functions.

One of the main sets of functions proposed for the extended face-processing system lie within the emotional domain. We found the face patches to be connected with the amygdala, orbitofrontal cortex, and the insula, three structures implicated in the processing of emotions, which, in humans, have been shown to be involved in evaluating faces on social dimensions such as trustworthiness [55]. The amygdala in particular also processes facial expression and gaze direction [56], two of the most important facial cues for social interactions. The orbitofrontal cortex, tightly interconnected with the amygdala, is thought to support the assignment of valence [57]. Thus, functional links between the face patches and core structures of the emotional brain exist, which may serve the utilization of facial information for the generation of emotional responses.

The fourth main set of connections we observed linked the face patch system, and in particular AM, the area at the top of the face-processing hierarchy [30], to structures supporting long-term memory, notably entorhinal cortex, the hippocampal formation, and the claustrum. The latter has been shown to be heavily connected to the temporal lobe and has been hypothesized to act as a relay for sensory inputs to mediotemporal memory areas [58]. Mediotemporal areas, including the hippocampus, contain face-selective cells thought to encode episodic memories [59]. Thus, FC of the most anterior–ventral face area to mediotemporal lobe structures exists, possibly supporting the encoding and retrieval of memories of familiar individuals. Taken together, we find evidence for the core face-processing system to be functionally connected to areas that are known to support cognitive, emotional, communicative, and mnemonic functions. Macaques thus appear to possess an extended face-processing system as proposed for humans [6].

### Comparison to rsfMRI Connectivity of the Human Face-Processing System

Neuroimaging studies investigating FC of human face-processing areas have found connectivity patterns that are broadly consistent with those we obtained in the macaque. In both species, correlations between the more posterior face-processing regions (the occipital face area (OFA) and the fusiform face area (FFA) in humans, and MF, ML, AF, and AL in the monkey) are stronger than with more anterior face-processing regions [60]. FC outside the core face-processing system also displays a similar pattern in monkeys and humans, with overlap in the occipital, temporal, and frontal lobes [61,62], as well as subcortically, including the hippocampus, amygdala, caudate nucleus, and thalamus [63]. Thus, internal and external FC of core face-processing areas is similar across the two primate species, consistent with the hypothesis of a deep evolutionary heritage of human face recognition abilities. The core face-processing systems in humans and monkeys are composed of multiple face areas. Establishing their homologies based on criteria like relative location and functional specialization [12] has proven difficult. Connectivity is a third, strong, and independent criterion for homology. The differences in face patch connectivity we found, in particular with mediotemporal lobe areas and the insula (S9 Fig), provide clear predictions for human combined fMRI/rsfMRI studies that localize all major face areas and map their FC. Currently available data on human face area connectivity, although not entirely consistent, points to a differentiation between dorsal and ventral face areas. Specifically, it has been found that the posterior STS displays stronger FC to premotor cortex than ventral face-processing areas, in particular the FFA [61,64] (but see [62]), in line with the finding that the STS face areas, but not the FFA, show structural connectivity to the ventrolateral prefrontal cortex [65]. If this differentiation between dorsal and ventral face areas is confirmed, it would indicate homology of the entire macaque face-processing network with the human dorsal face areas, a quite radical view that has previously been put forward on other grounds [11]. More data, in particular from the more variable human brain, will be needed to fully exploit the potential that these FC patterns hold in establishing homologies. Differential FC of the macaque face patches as we found hints at functional differentiations within the system and specialized roles these areas might play in social behavior. Comparing human and monkey rsfMRI connectivity networks to establish homologies as illustrated for the case of face areas is an approach that can be taken even further to understand the networks supporting complex cognitive functions.

### Substrates of High-Level Social Cognition with a Deep Evolutionary Heritage

It has been a long-standing question whether certain high-level sociocognitive skills, e.g., the ability to reason about the contents of other persons’ mental states, are uniquely human [15,18]. For example, monkeys do not infer the belief that someone has about his/her own state of mind, something humans do routinely [66]. There is behavioral evidence that monkeys display basic forms of TOM such as understanding what others, including human agents, see or know [17]. However, even this interpretation remains contested, since such behavior may also arise from reasoning about the observable behavior of others without explicitly representing the others’ mental state, i.e., without a TOM [20]. Even more so, there is uncertainty about the neural basis that supports high-level social cognition. A case in point is human area TPJ, for which an old-world monkey homolog has been outrightly rejected [67], or proposed to reside either in parietal area 7a [68] or the posterior STS [69]. While a whole battery of tasks is available to characterize high-level social cognition in humans, it has remained difficult to study the sociocognitive abilities of nonhuman primates experimentally. Here, we bypassed this issue and used a novel mapping strategy that utilizes the overlap of social brain areas and the DMN to identify putative homologs of human high-level social cognition systems. In humans, the main overlap between these two networks localizes to areas TPJ, PPC, and dmPFC. These areas have been strongly implicated in high-level social cognition, e.g., TOM in area TPJ and PPC, as well as the understanding of triadic interactions in the dmPFC [15,70,71]. We now find that a similar overlap between FPRSNs and DMN exists in the macaque. The overlap is specific to FPRSNs and prominently includes area TPO in the dorsal posterior STS where the human TPJ resides. Little is known about the role of TPO in social cognition, but it shares several functional characteristics with human TPJ: Like TPJ, TPO is a polysensory area [72] that responds to biological motion [73] and action observation [74] and is involved in attention [75,76]. Together with the connectivity overlap we observed, this suggests that a TPJ precursor or homolog exists in the macaque dorsal posterior STS and where it exists. Interestingly, this area is distinct from a more anterior STS region that has been shown to correlate with social network size [77].

Furthermore, we also find face-patch-specific overlap in areas 9M/10 in the dmPFC and areas PGm/23 in the medial PPC, which are well-established components of the human social brain and anatomically homologous in both species. Electrophysiological recordings during action- and error-monitoring of others [78,79] implicate the dmPFC in at least some aspects of social cognition in the monkey, which may form a precursor for the high-level sociocognitive functions that are supported by the dmPFC in humans [70]. Furthermore, the human dmPFC is also involved in more basic forms of social processing that explicitly rely on facial information, such as gaze following [80], possibly a consequence of functional integration with face processing. The medial PPC, which has been linked to understanding social interactions [81], inferring other people’s thoughts [82], and attributing mental states to others [83] in humans, has been shown to be active during action observation in monkeys [84], a basic ingredient for understanding the intentions of others. Thus, in addition to location and connectivity overlap, the functional properties of these areas are suggestive of a role in social cognition in the macaque.

Fig 5c also shows overlap around the occipitotemporal sulcus, including parts of areas V4, TFO, and TEO. In contrast to the other three regions, overlap in this area was not face-specific and connectivity to an STS object patch was even stronger than to a nearby face patch. This region has been found to be part of the human DMN [85] and is known to be anatomically connected both to the STS [45] and medial PCC [86,87], where our seed regions were located. Thus, connections exist that link this location to the DMN and face processing, rendering overlap in this region plausible. However, they do not suggest a specialized role of posteroventral cortex in social cognition.

Taken together, we find a pattern of overlap between the FPRSN and the DMN that includes the very areas that are selectively active in humans conducting the complex mental operations of TOM in macaque monkeys at rest. Hence, tapping into the social brain via an easily accessible sensory route uncovers similar regions as going through explicitly social cognition tasks in humans. This offers the exciting possibility to uncover the neural computations underlying sociocognitive operations. Since this overlap is present even under anesthesia, it is unlikely to reflect “mentalizing” as a default mode of processing [23], but it suggests a connectivity basis upon which a social default mode could have arisen. Our results thus point to a deep evolutionary heritage of a brain network composed of at least three areas for high-level social cognition.

### Conclusions

We used fMRI and rsfMRI to noninvasively assess FC within the face patch system and the embedding of the face patches into larger brain networks. Our results demonstrate that face-processing areas interconnect with each other and with a set of nonface areas involved in cognitive, emotional, and mnemonic functions, forming an extended face-processing network. Importantly, we can also show that this extended network seeded in the face patches exhibits a similar overlap with the DMN as areas involved in high-level social cognition in humans, which allows us to localize a putative TPJ homolog in the macaque STS. This suggests that the face patch system offers an easily accessible, sensory venue into studying the social brain in monkeys, and thus into the evolution of possibly uniquely human social skills.

## Materials and Methods

### Subjects

All animal procedures met the National Institutes of Health *Guide for Care and Use of Laboratory Animals*, and were approved by the local Institutional Animal Care and Use Committees of The Rockefeller University (protocol number 12585-H) and Weill-Cornell Medical College (protocol number 2010–0029), where MR scanning was performed. Data were acquired in six male, pair-housed macaque monkeys (5 *Macaca mulatta*, 1 *M*. *fascicularis*, 5.4–7.3 kg, age 3–5 yr).

### Surgery

Implantation of MR-compatible headposts (Ultem; General Electric Plastics), MR-compatible ceramic screws (Rogue Research), and acrylic cement (Grip Cement, Caulk; Dentsply International, and/or Palacos, Heraeus Kulzer GmbH) followed standard anesthetic, aseptic, and postoperative treatment protocols [88].

### Stimuli and Task

To localized face-selective ROIs, we used a standard face localizer [3]. In short, subjects fixated on a white dot at the center of the screen while we presented images of human and/or monkey faces, human and/or monkey body parts and/or headless bodies, manmade objects, and fruits, intermixed with baseline periods in which only the fixation dot was shown in a block design. Each block lasted 24–30 s. Fluid reward was delivered after variable periods of time (2–4 s), during which the subject maintained fixation within 2 degrees of the fixation dot. Only runs in which the subjects reached at least 90% fixation stability were used for analyses. Visual stimulation and reward were controlled using in house software (Visiko, M. Borisov). Stimuli were projected on a back-projection screen using a video projector (NEC NP3250, refresh rate 60 Hz, resolution 1024 × 768 pixel) with a custom lens. Eye position was measured at 120 Hz using a commercial eye monitoring system (ISCAN).

### Magnetic Resonance Imaging

Data were acquired on a 3 T scanner (Siemens TIM Trio). Functional data were acquired with an AC88 gradient insert (Siemens) and a custom 8-channel phased-array receive surface coil with a horizontally oriented single loop transmit coil (L. Wald, MGH/HST Martinos Center for Biomedical Imaging) while the monkeys were in sphinx position. Before scanning, the contrast agent ferumoxytol (8–10 mg of Fe per kg body weight) was injected into the femoral vein to increase the signal-to-noise ratio (SNR). For the face localizer experiments, we acquired between 16 and 51 runs of functional (*T*_2_*-weighted) gradient-echo echoplanar imaging (EPI) data per animal. Each run consisted of 196 volumes of 54 horizontally oriented slices (field of view [FOV] 96 mm, voxel size 1 × 1 × 1 mm, repetition time [TR] = 2 s, echo time [TE] = 16 ms, echo spacing [ESP] = 0.63 ms, bandwidth [BW] = 1,860 Hz/Px, flip angle [FA] = 80°, no gap) acquired in interleaved order with phase partial Fourier 7/8, and two times generalized autocalibrating partially parallel acquisitions (GRAPPA) acceleration, covering the whole brain. Additionally, we obtained field maps that allowed subsequent EPI undistortion [89]. For the resting state scans, we acquired 12 runs of 300 volumes of EPI data per animal, using the same sequences as in the localizer experiments. After induction with ketamine and dexmedetomidine hydrochloride, monkeys were lightly anesthetized with isoflurane (0.5%–0.6%) and placed in an MR-compatible monkey chair. The use of anesthesia follows the original definition of the monkey DMN [25] and conferred several technical advantages, including the elimination of motion artifacts and the ability to record cardiac and respiratory signals. Although anesthesia can affect systemic physiology, neural activity, vasoactive signal transmission and/or vascular reactivity [90], it has been shown that anesthesia preserves the correlation structure that is observed when the subjects are awake [91,92], that significant changes in correlation patterns occur only under much deeper levels of isoflurane anesthesia (>1.5%) [93] than the one we used (0.5%–0.6%), and that anesthetized monkey resting state networks, including the DMN, are strikingly similar to the same networks observed in awake humans [25,94]. Electrocardiogram (sampling rate 400 Hz) and breathing rate (sampling rate 50 Hz) were acquired together with the imaging data. Anatomical images were obtained in a separate session using a *T*_*1*_-weighted magnetization-prepared rapid gradient echo (MPRAGE) sequence (FOV 128 mm, voxel size 0.5 × 0.5 × 0.5 mm, TR = 2.53 s, TE = 3.07 ms, ESP = 7.3 ms, BW = 190 Hz/Px, FA = 7°, 240 slices) and a custom 1-channel receive coil (L. Wald, MGH/HST Martinos Center for Biomedical Imaging) while the monkeys were anesthetized (isoflurane 1.5%–2%) and positioned in an MR-compatible stereotactic frame (Kopf Instruments).

### Analyses

Data were analyzed in Freesurfer (v5.1, https://surfer.nmr.mgh.harvard.edu) and Matlab (R2011b, The Mathworks) using custom code. The first five volumes of each functional run were excluded to prevent *T*_1_ saturation effects. Preprocessing included slice scan time correction, motion correction, and geometric distortion correction by means of a field map. Outliers in the time courses were detected semiautomatically based on a threshold of median absolute deviation = 3.5 [95] in the mean whole-brain time course and later excluded from analyses. To create inflated cortical surface reconstructions, the gray–white matter boundary in the skull-stripped anatomical scans was segmented, reconstructed, smoothed, and inflated separately for each hemisphere [96,97].

#### Localization of face patches

In each animal and each hemisphere, we identified five temporal (ML, MF, AL, AF, AM) face patches and one frontal (PO) face patch based on the functional localizer, following established procedures [2]. Data were slightly smoothed with a Gaussian kernel (2 mm full-width at half-maximum), whitened using a first-order autoregressive model, and detrended for first and second order polynomials. For each animal, we calculated a GLM with the stimulation conditions as predictors as well as six orthogonalized nuisance regressors accounting for motion artifacts. As in previous studies, face patches were identified based on anatomical location and relative position [2] in uncorrected significance maps (ranging between *p* < 0.05 and *p* < 10^−110^) resulting from the contrasts (faces versus objects and bodies). Whenever possible, homolog face patches were then joined into bilateral ROIs for further processing. This way, we were able to identify six face patch ROIs in all six animals.

#### Analysis of resting state data

To analyze the FPRSNs, we aligned the functional data from the localizer scans with the resting state scans. We then extracted seed time courses in the resting state data from the face patch ROIs in volume space as the first Eigenvariate (using the mean time course instead gave almost identical results). To remove the effects of heartbeat and respiration [98], we created nuisance regressors from electrocardiographic (EKG) and breathing rate measurements acquired during the scans using the RETROICOR algorithm [99] as implemented in the PhLEM toolbox [100]. Runs for which the EKG and/or respiration data were incomplete or contained many artifacts (e.g., because the recording devices detached during the measurement) were removed from the analyses (<10% of the data). Additionally, MRI data were high-pass filtered at 0.0025 Hz and detrended for first and second order polynomials. Global mean regression was not performed. For ROI analyses, seed time courses were extracted from the unsmoothed data after regressing out the effects of motion, heartbeat, and respiration. Correlations between residuals were Fisher *z*-transformed per run, averaged, and back transformed to yield one *r* value per subject; this *r* value was then *z*-transformed and tested against 0 over subjects, using one-sided Wilcoxon signed rank tests (we used one-sided tests whenever we had a hypothesis about the directionality of the effects, and two-sided tests otherwise). Average *r* values over subjects were obtained by averaging and back transforming the per-subject *z*-values [101]. For median differences, we report 95% confidence intervals for the Hodges-Lehman estimator, a rank-based, unbiased estimator of the median [102]. Whole-brain analyses were performed in surface and in volume space. For surface-based analyses, functional data were aligned to an average anatomical surface template based on five subjects in the study (see below). Data was smoothed on the surface with a 1.25 mm kernel, thus preserving anatomical specificity for tissue type and anatomical location. To identify the cortical areas that show resting state connectivity with the face patches, we ran a FFX GLM for each hemisphere on the surface template with the respective face patch time course as a predictor, as well as nuisance regressors for motion (6), heartbeat (4), and respiration (10). To attenuate the effects of individual subjects on the group results, we used the same number of runs from each animal (8, i.e., 80 min of data). We then contrasted the respective face patch predictor against 0 to obtain *p*-value maps of connected voxels (two-tailed *t* test). Cluster size thresholding based on 10,000 permutations of voxel locations assuming a *z*-distribution was used to correct for multiple comparisons [103]. The cluster-forming threshold was *p* < 0.00001 for AF, AL, MF, ML, and PO, and *p* < 0.05 for AM. AM was analyzed at a more lenient statistical threshold because it had a lower SNR but nevertheless showed a very similar pattern of results to the other face patches, including FC to lateral prefrontal, temporal, parietal, and occipital areas. Conjunction maps [104] were calculated from uncorrected maps of AF, AL, MF, ML, and PO and then corrected for multiple comparisons using cluster size thresholding (*p* < 0.05, cluster-forming threshold *p* < 0.001, 10,000 permutations). Data were deposited in the Dryad repository: http://dx.doi.org/10.5061/dryad.80476 [39]. For volume-based analyses, data were nonlinearly aligned to an average volume template (see below) using JIP (http://www.nmr.mgh.harvard.edu/~jbm/jip/) and smoothed using a 2 mm Gaussian kernel. The further analysis steps were the same as in surface space.

To assess the specificity of face patch FC, we first identified voxels activated by manmade objects in the localizer scan from the contrast (objects versus faces, bodies, and fruits), analogously to how we identified the face patches. We isolated an object-selective patch in the anterior lip of the STS in each animal and hemisphere, approximately 4 mm posterior to the face patch AL, as previously described [105]. Before extracting seed time courses in the resting state data from the object patch ROIs as the first Eigenvariate, we matched the number of voxels in the object patch to the number of voxels in AL for each animal and hemisphere, respectively, and then joined homolog object ROIs into one bilateral ROI per animal. Next, to avoid issues of multi-colinearity, we separately orthogonalized the time course of the object patch ROI and the time course of AL against the global brain-wide mean, using the Gram-Schmidt algorithm [106]. This preserves variance that is specific to the respective ROI and effectively reduced the correlation between face patch and object patch predictors. We then ran a FFX GLM for each hemisphere in surface space with the AL and the object patch time courses as predictors, as well as nuisance regressors for motion (6), heartbeat (4), and respiration (10). Finally, we contrasted the respective face patch predictor against the object patch predictor. The resulting maps of differential face patch versus object patch connectivity were corrected for multiple comparisons using cluster size thresholding (*p* < 0.05, cluster-forming threshold *p* < 10^−2^, 10,000 permutations). To further assess the relationship between face selectivity and connectivity, we also compared the connectivity of each face patch to face- and object-selective voxels in volume space, at matched levels of category selectivity. To this end, we first calculated *d’* between faces and objects as a measure of selectivity for each voxel in each hemisphere from the localizer data sets. *d’* was defined as

$$d\prime \;=\;\frac{M(f)-M(nf)}{\sqrt{\frac{\sigma {\left(f\right)}_{}^{2}+\sigma {\left(nf\right)}_{}^{2}}{2}}}$$

where *M(f)* and *M(nf)* are the mean response to the face and nonface category with the highest mean response, respectively, and *σ* ^*2*^ *(f)* and *σ* ^*2*^ *(nf)* are their variances, taken from the first level GLM. Because rsfMRI connectivity is known to fall off with distance [107,108], we also calculated the Euclidean distance from the voxel with peak selectivity within each respective face patch to the remaining voxels within the same hemisphere. Euclidean distance is a good approximation to more refined distance measures, e.g., measures based on tractography [109]. Subsequently, for each face-selective voxel (*d’* ≥ 2) outside the face patch under consideration (AF, AL, MF, ML, PO), we searched for the object-selective voxel that was the closest match both in its degree of selectivity as well as in distance from the face patch. To this end, we first considered the differences between each face-selective voxel and all object-selective voxels in selectivity and distance, respectively, as XY coordinates; we then found the coordinates that were closest to the origin (0,0—no difference); the object-selective voxel with the shortest Euclidean distance (<0.3) in this hypothetical XY space was chosen for the pair. This procedure assured that pairs of face- and object-selective voxels were matched as closely as possible, weighting selectivity and distance equally. We confirmed that across hemispheres and face patches, this led to no significant difference between face and object selectivity for 50 out of 54 patch × hemisphere combinations, and to no significant difference in distance for 51 out of 54 patch × hemisphere combinations, using two-sided *t* tests at *p* < 0.05. Finally, we compared the connectivity (*z*-transformed correlation coefficients, corrected for multiple comparisons using the FDR [110] at *q* = 0.01) for matched face and object-selective voxels across 12 hemispheres for AF, AL, MF, ML, and six hemispheres for PO using paired, one-sided *t* tests. Thus, only differences in connectivity strength for matched pairs entered the analysis. These analyses were done in native volume space on slightly smoothed (2 mm Gaussian kernel) data. To assess the overlap with the DMN, we placed an additional seed in PPC (anatomically defined area 31/PGm in the left and right hemisphere, 46 voxels) of each monkey, following the original definition of the monkey DMN [25] and previous studies assessing the overlap between the DMN and the social brain in humans (e.g., [28]). DMN resting state connectivity maps were then calculated as described above. To further quantify the overlap between each FPRSN and the DMN, we calculated the Jaccard Index (# of voxels intersection / # of voxels union) [111] for each pair of networks over a range of statistical thresholds (*p* < 0.05, 10^−3:-1:-90^). Statistical significance of the overlap was assessed by generating *z*-distributed random noise fields which had the same smoothness as the DMN inside a brain mask; at each threshold, we created 5,000 such maps per face patch, thresholded them to the same number of significant voxels as the original DMN map, and computed the overlap of each noise map with the respective face patch resting state map. We then compared the empirically observed Jaccard Index to the thus obtained distribution of Jaccard Indices arising by chance, which yielded a *p*-value. To correct for multiple comparisons (face patches × thresholds), we used a FDR at *q* = 0.01. Finally, conjunction maps were computed for illustration purposes. To assess the specificity of overlap between FPRSN and the DMN, we determined the overlap between the resting state network of the object patch we identified in the anterior lip of the STS and the DMN. To assess whether there was a difference in the amount of overlap with the DMN, we then compared the ratio of overlapping to nonoverlapping DMN voxels between the AL FPRSN and the object patch resting state network using a *χ* ^*2*^-test for the same range of *p*-value thresholds as before (*p* < 0.05, 10^−3:-1:-90^). Finally, we also compared the strength of connectivity to the object patch in voxels for which AL showed significant overlap with the DMN, contrasting AL with the object patch in a FFX GLM. The resulting maps were corrected for multiple comparisons (the number of voxels in which AL showed overlap with the DMN) using a FDR at *q* = 0.05. All overlap analyses were done in volume space after alignment to the average volume template.

#### Generation of anatomical templates for group analyses

In order to conduct group statistics over the whole brain, functional data had to be brought into a common space. To this end, we created an anatomical surface template for alignment and visualization of the results. This was done by iteratively aligning and averaging the inflated surfaces of the five *M*. *mulatta* in the study using Freesurfer [112]. This average surface was also brought into F99 space [113] using landmark surface-based registration [114] in Caret (v5.65; http://www.nitrc.org/projects/caret/) for use with surface-based atlases. Additionally, we created a volume template in AFNI (http://afni.nimh.nih.gov) for volume-based group analyses. This template was aligned to the MNI-Paxinos macaque template brain [38,115].

## Supporting Information

S1 Fig Correlation between rsfMRI and microstimulation connectivity. Spearman rank correlations revealed a strong correlation (*r* = 0.5256, *p* = 0.0365, red) between the connection strengths obtained through noninvasive rsfMRI and electrical microstimulation [3]. After the exclusion of one extreme data point (AL–AF), the rank correlation rose to *r* = 0.6314, *p* = 0.0116 (blue). The same results were obtained using the robust correlation method Shepherd’s Pi (*r* = 0.6352, *p* = 0.0219) [33]. To bring rsfMRI and microstimulation connectivity onto the same scale, we first computed the median connectivity strength across animals per connection for the microstimulation data and then computed tied ranks within the columns of this and the resting state connectivity matrices.
(PDF)

S2 Fig Cortical FC of the face patch PL. PL, which could be identified in four out of six animals in the study, showed a connectivity pattern (multiple comparisons corrected using cluster size thresholding at *p* < 0.05) that was similar to that of the other face patches, including connectivity to prefrontal, premotor, and occipitotemporal areas. Results are shown on inflated and flattened left and right hemispheres in F99 space. Representative locations of the face patches are outlined in black. For comparison, three broad networks of connectivity from the main conjunction analysis (including AF, AL, MF, ML, and PO) are highlighted: areas in prefrontal cortex (light blue), a premotor-parietal network (blue), and an occipitotemporal network including ventral stream areas. Areal boundaries are from Lewis & van Essen [35]. Data shown here are publicly available at the Dryad Digital Repository [39].
(PDF)

S3 Fig Cortical FC of the face patches. The central panel shows the results of a conjunction analysis of the maps from AF, AL, MF, ML, and PO (multiple comparisons corrected using cluster size thresholding at *p* < 0.05) on an inflated and flattened left hemisphere in F99 space. Highlighted are three broad networks of connectivity: areas in prefrontal cortex (light blue), a premotor-parietal network (blue), and an occipitotemporal network including ventral stream areas (green). The surrounding panels show connectivity maps of the individual face patches (multiple comparisons corrected using cluster size thresholding at *p* < 0.05), along with the areal boundaries of the conjunction analysis (light blue, blue, green). Representative locations of the face patches are outlined in black. Areal boundaries are from Lewis & van Essen [35]. See S1 Table for a list of area names. Data shown here are publicly available at the Dryad Digital Repository [39].
(PDF)

S4 Fig Complete areal labeling of face patch FC in the right hemisphere based on Lewis & van Essen [35]. Shown are the results of a conjunction analysis of the maps from AF, AL, MF, ML, and PO (multiple comparisons corrected using cluster size thresholding at *p* < 0.05) on an inflated and flattened right hemisphere in F99 space. Representative locations of the face patches are outlined in black. Data shown here are publicly available at the Dryad Digital Repository [39].
(PDF)

S5 Fig Alternative partitioning scheme for the premotor-parietal FC based on Paxinos et al. [38]. Shown are the results of a conjunction analysis of the maps from AF, AL, MF, ML, and PO (multiple comparisons corrected using cluster size thresholding at *p* < 0.05) on an inflated and flattened left hemisphere in F99 space. Representative locations of the face patches are outlined in black. Data shown here are publicly available at the Dryad Digital Repository [39].
(PDF)

S6 Fig Number of face patches connected to a given vertex. For each vertex, we quantified how many face patches were functionally connected after correction for multiple comparisons using cluster size thresholding at *p* < 0.05 (cf. outer panels of Fig 2 & S3 Fig). Results are shown on inflated and flattened left and right hemispheres in F99 space. Representative locations of the face patches are outlined in black/white. Data shown here are publicly available at the Dryad Digital Repository [39].
(PDF)

S7 Fig Cortical FC of the temporal face patches. Shown are the results of a conjunction analysis of the maps from AF, AL, MF, and ML (multiple comparisons corrected using cluster size thresholding at *p* < 0.05) on inflated and flattened left and right hemispheres in F99 space. Highlighted are three broad networks of connectivity from the main conjunction analysis including the orbitofrontal face patch PO: areas in prefrontal cortex (light blue), a premotor-parietal network (blue), and an occipitotemporal network including ventral stream areas (green). Areal boundaries are from Lewis & van Essen [35]. Representative locations of the face patches are outlined in black. Data shown here are publicly available at the Dryad Digital Repository [39].
(PDF)

S8 Fig FC of the FPRSN in volume space. Shown are the results of a conjunction analysis (uncorrected) of the rsfMRI maps of bilateral face patches AF, AL, MF, ML, and PO, overlaid on coronal slices of the MNI-Paxinos template brain, in radiological convention (left is right). Coordinates are relative to the center of the anterior commissure. Area labels are based on Paxinos et al. [38].
(PDF)

S9 Fig Differences in FC between the face patches. (a) connectivity to the insula was more prominent for AL, ML, and PO than for MF and AF; (b) AL and AF connectivity extended more posteriorly on the dorsolateral surface towards the central sulcus than any of the other face patches (the premotor-parietal network from the main conjunction analysis is shown for reference); (c) only AM showed connectivity to medial temporal lobe structures (entorhinal cortex, perirhinal cortical areas 35 and 36). All contrasts were corrected for multiple comparisons using a FDR at *q* = 0.05. Results are shown on an inflated and flattened right hemisphere in F99 space, negative differences are truncated for display purposes. Representative locations of the face patches are outlined in black. Areal boundaries are from Lewis & van Essen [35]. Data shown here are publicly available at the Dryad Digital Repository [39].
(PDF)

S10 Fig Overlap analysis of DMN and the FPRSN of AM. (a) Voxels in area TPO in the dorsal bank of the posterior STS that show significant connectivity both with the PPC and AM at *p* < 10^−3.5^, uncorrected. (c) Voxels in dmPFC and medial PPC that show significant connectivity both with the PPC and AM at *p* < 10^−1.6^, uncorrected. The overlap between the resting state network of AM and the DMN was highly consistent with that of the other face patches, localizing to three areas known to support high-level social cognition in humans. Results are overlaid on the MNI-Paxinos template brain. Coordinates are relative to the center of the anterior commissure.
(PDF)

S11 Fig Overlap between the DMN and the FPRSNs, Jaccard Indices. a) Results of the permutation tests for significant overlap between the DMN and individual FPRSNs, quantified as Jaccard Indices, over a wide range of statistical thresholds. The blue lines show the empirically observed overlap, while the green lines show the average degree of overlap between the respective face patch map and 5,000 randomly generated noise maps with spatial smoothness and number of significant voxels matched to those of the DMN map at each threshold. b) After correction for multiple comparisons (FDR, *q* = 0.01), there was significant overlap between the DMN and each of the FPRSNs until thresholds were so conservative that the likelihood of overlap was minimized.
(PDF)

S12 Fig Specificity of overlap between the DMN and the FPRSNs. We compared the overlap between the resting state networks of the face patch AL and the DMN to the overlap between the resting state networks of a nearby object patch and the DMN. The ratio of Jaccard Indices for AL-DMN to object patch-DMN rapidly increases as statistical thresholds get more conservative. For most thresholds tested, there was no overlap between the object patch resting state network and the DMN (orange area). For the remaining thresholds, *χ* ^*2*^-tests of the ratio of overlapping to nonoverlapping DMN voxels between AL and the object patch showed that face patch connectivity overlap always exceeded object patch connectivity overlap (corrected for multiple comparisons using a FDR at *q* = 0.01).
(PDF)

S1 Table Areas left hemisphere. Area names are from Lewis & van Essen [35].
(DOC)

## Abbreviations

AF: anterior fundus
AL: anterior lateral
AM: anterior medial
DMN: default mode network
dmPFC: dorsomedial prefrontal cortex
FC: functional connectivity
FDR: false discovery rate
FFA: fusiform face area
FFX: fixed effects
FPRSN: face patch resting state network
GLM: general linear model
MF: middle fundus
ML: middle lateral
OFA: occipital face area
PL: posterior lateral
PO: prefrontal orbital
PPC: posterior parietal cortex
ROI: region of interest
rsfMRI: resting state functional magnetic resonance imaging
SEF: supplementary eye field
STS: superior temporal sulcus
TOM: theory of mind
TPJ: temporoparietal junction

## References

[1] Allison T, Puce A, McCarthy G. Social perception from visual cues: role of the STS region. Trends in cognitive sciences. 2000;4(7):267–78. Epub 2000/06/22. 10.1016/s1364-6613(00)01501-1 10859571

[2] Tsao DY, Moeller S, Freiwald WA. Comparing face patch systems in macaques and humans. Proceedings of the National Academy of Sciences of the United States of America. 2008;105(49):19514–9. Epub 2008/11/27. 10.1073/pnas.0809662105 19033466 PMC2614792

[3] Moeller S, Freiwald WA, Tsao DY. Patches with links: a unified system for processing faces in the macaque temporal lobe. Science. 2008;320(5881):1355–9. Epub 2008/06/07. 10.1126/science.1157436 18535247 PMC8344042

[4] Deaner RO, Platt ML. Reflexive social attention in monkeys and humans. Current biology: CB. 2003;13(18):1609–13. Epub 2003/09/19. 10.1016/j.cub.2003.08.025 13678591

[5] Crouzet SM, Kirchner H, Thorpe SJ. Fast saccades toward faces: face detection in just 100 ms. Journal of vision. 2010;10(4):16 1–7. Epub 2010/05/15. 10.1167/10.4.16 20465335

[6] Haxby JV, Hoffman EA, Gobbini MI. The distributed human neural system for face perception. Trends in cognitive sciences. 2000;4(6):223–33. Epub 2000/05/29. 10.1016/s1364-6613(00)01482-0 10827445

[7] Smith SM, Vidaurre D, Beckmann CF, Glasser MF, Jenkinson M, Miller KL, et al Functional connectomics from resting-state fMRI. Trends in cognitive sciences. 2013;17(12):666–82. Epub 2013/11/19. 10.1016/j.tics.2013.09.016 24238796 PMC4004765

[8] Miranda-Dominguez O, Mills BD, Grayson D, Woodall A, Grant KA, Kroenke CD, et al Bridging the gap between the human and macaque connectome: a quantitative comparison of global interspecies structure-function relationships and network topology. The Journal of neuroscience: the official journal of the Society for Neuroscience. 2014;34(16):5552–63. Epub 2014/04/18. 10.1523/JNEUROSCI.4229-13.2014 24741045 PMC3988411

[9] Behrens TE, Sporns O. Human connectomics. Current opinion in neurobiology. 2012;22(1):144–53. 10.1016/j.conb.2011.08.005 21908183 PMC3294015

[10] Hutchison RM, Everling S. Monkey in the middle: why non-human primates are needed to bridge the gap in resting-state investigations. Frontiers in neuroanatomy. 2012;6:29 Epub 2012/08/03. 10.3389/fnana.2012.00029 22855672 PMC3405297

[11] Ku SP, Tolias AS, Logothetis NK, Goense J. fMRI of the face-processing network in the ventral temporal lobe of awake and anesthetized macaques. Neuron. 2011;70(2):352–62. Epub 2011/04/28. 10.1016/j.neuron.2011.02.048 21521619

[12] Yovel G, Freiwald WA. Face recognition systems in monkey and human: are they the same thing? F1000prime reports. 2013;5:10 Epub 2013/04/16. 10.12703/P5-10 23585928 PMC3619156

[13] Brothers L. The neural basis of primate social communication. Motiv Emot. 1990;14(2):81–91. 10.1007/bf00991637

[14] Vernon RJ, Sutherland CA, Young AW, Hartley T. Modeling first impressions from highly variable facial images. Proceedings of the National Academy of Sciences of the United States of America. 2014;111(32):E3353–61. 10.1073/pnas.1409860111 25071197 PMC4136614

[15] Saxe R. Uniquely human social cognition. Current opinion in neurobiology. 2006;16(2):235–9. Epub 2006/03/21. 10.1016/j.conb.2006.03.001 16546372

[16] Call J, Tomasello M. Does the chimpanzee have a theory of mind? 30 years later. Trends in cognitive sciences. 2008;12(5):187–92. Epub 2008/04/22. 10.1016/j.tics.2008.02.010 18424224

[17] Drayton LA, Santos LR. A decade of theory of mind research on Cayo Santiago: insights into rhesus macaque social cognition. American journal of primatology. 2014 Epub 2015/01/06. 10.1002/ajp.22362 25556543

[18] Rushworth MF, Mars RB, Sallet J. Are there specialized circuits for social cognition and are they unique to humans? Current opinion in neurobiology. 2013;23(3):436–42. Epub 2013/01/08. 10.1016/j.conb.2012.11.013 23290767

[19] Call J, Santos LR. Understanding other minds In: Mitani JC, Call J, Kappeler PM, Palombit RA, Silk JB, editors. The evolution of primate societies. Chicago: The University of Chicago Press; 2012 p. 664–81.

[20] Penn DC, Povinelli DJ. On the lack of evidence that non-human animals possess anything remotely resembling a 'theory of mind'. Philosophical transactions of the Royal Society of London Series B, Biological sciences. 2007;362(1480):731–44. Epub 2007/02/01. 10.1098/rstb.2006.2023 17264056 PMC2346530

[21] Kumar S, Hedges SB. A molecular timescale for vertebrate evolution. Nature. 1998;392(6679):917–20. Epub 1998/05/15. 10.1038/31927 9582070

[22] Corbetta M, Patel G, Shulman GL. The reorienting system of the human brain: from environment to theory of mind. Neuron. 2008;58(3):306–24. Epub 2008/05/10. 10.1016/j.neuron.2008.04.017 18466742 PMC2441869

[23] Schilbach L, Eickhoff SB, Rotarska-Jagiela A, Fink GR, Vogeley K. Minds at rest? Social cognition as the default mode of cognizing and its putative relationship to the "default system" of the brain. Consciousness and cognition. 2008;17(2):457–67. Epub 2008/04/25. 10.1016/j.concog.2008.03.013 18434197

[24] Mars RB, Neubert FX, Noonan MP, Sallet J, Toni I, Rushworth MF. On the relationship between the "default mode network" and the "social brain". Frontiers in human neuroscience. 2012;6:189 Epub 2012/06/28. 10.3389/fnhum.2012.00189 22737119 PMC3380415

[25] Vincent JL, Patel GH, Fox MD, Snyder AZ, Baker JT, van Essen DC, et al Intrinsic functional architecture in the anaesthetized monkey brain. Nature. 2007;447(7140):83–6. Epub 2007/05/04. 10.1038/nature05758 17476267

[26] Margulies DS, Vincent JL, Kelly C, Lohmann G, Uddin LQ, Biswal BB, et al Precuneus shares intrinsic functional architecture in humans and monkeys. Proceedings of the National Academy of Sciences of the United States of America. 2009;106(47):20069–74. Epub 2009/11/12. 10.1073/pnas.0905314106 19903877 PMC2775700

[27] Buckner RL, Andrews-Hanna JR, Schacter DL. The brain's default network: anatomy, function, and relevance to disease. Annals of the New York Academy of Sciences. 2008;1124:1–38. Epub 2008/04/11. 10.1196/annals.1440.011 18400922

[28] Amft M, Bzdok D, Laird AR, Fox PT, Schilbach L, Eickhoff SB. Definition and characterization of an extended social-affective default network. Brain structure & function. 2015;220(2):1031–49. Epub 2014/01/09. 10.1007/s00429-013-0698-0 24399179 PMC4087104

[29] Tsao DY, Schweers N, Moeller S, Freiwald WA. Patches of face-selective cortex in the macaque frontal lobe. Nature neuroscience. 2008;11(8):877–9. Epub 2008/07/16. 10.1038/nn.2158 18622399 PMC8123225

[30] Freiwald WA, Tsao DY. Functional compartmentalization and viewpoint generalization within the macaque face-processing system. Science. 2010;330(6005):845–51. Epub 2010/11/06. 10.1126/science.1194908 21051642 PMC3181095

[31] Seltzer B, Pandya DN. Intrinsic connections and architectonics of the superior temporal sulcus in the rhesus monkey. The Journal of comparative neurology. 1989;290(4):451–71. Epub 1989/12/22. 10.1002/cne.902900402 2482305

[32] Ban T, Naito J, Kawamura K. Commissural afferents to the cortex surrounding the posterior part of the superior temporal sulcus in the monkey. Neuroscience letters. 1984;49(1–2):57–61. Epub 1984/08/24. 10.1016/0304-3940(84)90136-8 6493598

[33] Schwarzkopf DS, De Haas B, Rees G. Better ways to improve standards in brain-behavior correlation analysis. Frontiers in human neuroscience. 2012;6:200 10.3389/fnhum.2012.00200 22811662 PMC3397314

[34] Sultan F, Augath M, Murayama Y, Tolias AS, Logothetis N. esfMRI of the upper STS: further evidence for the lack of electrically induced polysynaptic propagation of activity in the neocortex. Magnetic resonance imaging. 2011;29(10):1374–81. Epub 2011/07/16. 10.1016/j.mri.2011.04.005 21757310

[35] Lewis JW, Van Essen DC. Mapping of architectonic subdivisions in the macaque monkey, with emphasis on parieto-occipital cortex. The Journal of comparative neurology. 2000;428(1):79–111. 10.1002/1096-9861(20001204)428:1<79::aid-cne7>3.0.co;2-q 11058226

[36] Ó Scalaidhe SP, Wilson FA, Goldman-Rakic PS. Face-selective neurons during passive viewing and working memory performance of rhesus monkeys: evidence for intrinsic specialization of neuronal coding. Cerebral cortex. 1999;9(5):459–75. Epub 1999/08/18. 10.1093/cercor/9.5.459 10450891

[37] Rizzolatti G, Luppino G. The cortical motor system. Neuron. 2001;31(6):889–901. Epub 2001/10/03. 10.1016/s0896-6273(01)00423-8 11580891

[38] Paxinos G, Huang XF, Petrides M, Toga AW. The Rhesus monkey brain in stereotactic coordinates. 2nd ed London, UK: Elsevier Academic Press; 2008.

[39] Schwiedrzik CM, Zarco W, Everling S, Freiwald WA. Data from: Face patch resting state networks link face processing to social cognition. Dryad Digital Repository. 2015. Openly available via: 10.5061/dryad.80476 PMC4562659 26348613

[40] Nguyen MN, Hori E, Matsumoto J, Tran AH, Ono T, Nishijo H. Neuronal responses to face-like stimuli in the monkey pulvinar. The European journal of neuroscience. 2013;37(1):35–51. Epub 2012/11/06. 10.1111/ejn.12020 23121157

[41] Leonard CM, Rolls ET, Wilson FA, Baylis GC. Neurons in the amygdala of the monkey with responses selective for faces. Behavioural brain research. 1985;15(2):159–76. Epub 1985/04/01. 10.1016/0166-4328(85)90062-2 3994832

[42] Mahon BZ, Caramazza A. What drives the organization of object knowledge in the brain? Trends in cognitive sciences. 2011;15(3):97–103. Epub 2011/02/15. 10.1016/j.tics.2011.01.004 21317022 PMC3056283

[43] Saygin ZM, Osher DE, Koldewyn K, Reynolds G, Gabrieli JD, Saxe RR. Anatomical connectivity patterns predict face selectivity in the fusiform gyrus. Nature neuroscience. 2012;15(2):321–7. Epub 2011/12/27. 10.1038/nn.3001 PMC3267901 22197830

[44] Mantini D, Gerits A, Nelissen K, Durand JB, Joly O, Simone L, et al Default mode of brain function in monkeys. The Journal of neuroscience: the official journal of the Society for Neuroscience. 2011;31(36):12954–62. Epub 2011/09/09. 10.1523/JNEUROSCI.2318-11.2011 21900574 PMC3686636

[45] Kravitz DJ, Saleem KS, Baker CI, Ungerleider LG, Mishkin M. The ventral visual pathway: an expanded neural framework for the processing of object quality. Trends in cognitive sciences. 2013;17(1):26–49. Epub 2012/12/26. 10.1016/j.tics.2012.10.011 23265839 PMC3532569

[46] Johnson MH. Subcortical face processing. Nature reviews Neuroscience. 2005;6(10):766–74. Epub 2005/11/09. 10.1038/nrn1766 16276354

[47] Freedman DJ, Riesenhuber M, Poggio T, Miller EK. Categorical representation of visual stimuli in the primate prefrontal cortex. Science. 2001;291(5502):312–6. Epub 2001/02/24. 10.1126/science.291.5502.312 11209083

[48] Kaping D, Vinck M, Hutchison RM, Everling S, Womelsdorf T. Specific contributions of ventromedial, anterior cingulate, and lateral prefrontal cortex for attentional selection and stimulus valuation. PLoS biology. 2011;9(12):e1001224 Epub 2012/01/05. 10.1371/journal.pbio.1001224 22215982 PMC3246452

[49] Corbetta M, Shulman GL. Spatial neglect and attention networks. Annual review of neuroscience. 2011;34:569–99. Epub 2011/06/23. 10.1146/annurev-neuro-061010-113731 21692662 PMC3790661

[50] Harries MH, Perrett DI. Visual processing of faces in temporal cortex: physiological evidence for a modular organization and possible anatomical correlates. Journal of cognitive neuroscience. 1991;3(1):9–24. Epub 1991/01/01. 10.1162/jocn.1991.3.1.9 23964802

[51] Huerta MF, Kaas JH. Supplementary eye field as defined by intracortical microstimulation: connections in macaques. The Journal of comparative neurology. 1990;293(2):299–330. Epub 1990/03/08. 10.1002/cne.902930211 19189718

[52] Luppino G, Calzavara R, Rozzi S, Matelli M. Projections from the superior temporal sulcus to the agranular frontal cortex in the macaque. The European journal of neuroscience. 2001;14(6):1035–40. Epub 2001/10/12. 10.1046/j.0953-816x.2001.01734.x 11595042

[53] Takahara D, Inoue K, Hirata Y, Miyachi S, Nambu A, Takada M, et al Multisynaptic projections from the ventrolateral prefrontal cortex to the dorsal premotor cortex in macaques—anatomical substrate for conditional visuomotor behavior. The European journal of neuroscience. 2012;36(10):3365–75. Epub 2012/08/14. 10.1111/j.1460-9568.2012.08251.x 22882424

[54] Ferrari PF, Gallese V, Rizzolatti G, Fogassi L. Mirror neurons responding to the observation of ingestive and communicative mouth actions in the monkey ventral premotor cortex. The European journal of neuroscience. 2003;17(8):1703–14. Epub 2003/05/20. 10.1046/j.1460-9568.2003.02601.x 12752388

[55] Winston JS, Strange BA, O'Doherty J, Dolan RJ. Automatic and intentional brain responses during evaluation of trustworthiness of faces. Nature neuroscience. 2002;5(3):277–83. Epub 2002/02/19. 10.1038/nn816 11850635

[56] Hoffman KL, Gothard KM, Schmid MC, Logothetis NK. Facial-expression and gaze-selective responses in the monkey amygdala. Current biology: CB. 2007;17(9):766–72. Epub 2007/04/07. 10.1016/j.cub.2007.03.040 17412586

[57] Salzman CD, Fusi S. Emotion, cognition, and mental state representation in amygdala and prefrontal cortex. Annual review of neuroscience. 2010;33:173–202. Epub 2010/03/25. 10.1146/annurev.neuro.051508.135256 20331363 PMC3108339

[58] Gattass R, Soares JG, Desimone R, Ungerleider LG. Connectional subdivision of the claustrum: two visuotopic subdivisions in the macaque. Frontiers in systems neuroscience. 2014;8:63 Epub 2014/05/23. 10.3389/fnsys.2014.00063 24847219 PMC4019870

[59] Sliwa J, Plante A, Duhamel JR, Wirth S. Independent Neuronal Representation of Facial and Vocal Identity in the Monkey Hippocampus and Inferotemporal Cortex. Cerebral cortex. 2014;in press. Epub 2014/11/19. 10.1093/cercor/bhu257 25405945

[60] O'Neil EB, Hutchison RM, McLean DA, Kohler S. Resting-state fMRI reveals functional connectivity between face-selective perirhinal cortex and the fusiform face area related to face inversion. NeuroImage. 2014;92:349–55. Epub 2014/02/18. 10.1016/j.neuroimage.2014.02.005 24531049

[61] Zhang H, Tian J, Liu J, Li J, Lee K. Intrinsically organized network for face perception during the resting state. Neuroscience letters. 2009;454(1):1–5. Epub 2009/05/12. 10.1016/j.neulet.2009.02.054 19429043 PMC2702662

[62] Hutchison RM, Culham JC, Everling S, Flanagan JR, Gallivan JP. Distinct and distributed functional connectivity patterns across cortex reflect the domain-specific constraints of object, face, scene, body, and tool category-selective modules in the ventral visual pathway. NeuroImage. 2014;96C:216–36. Epub 2014/04/05. 10.1016/j.neuroimage.2014.03.068 24699018

[63] Turk-Browne NB, Norman-Haignere SV, McCarthy G. Face-specific resting functional connectivity between the fusiform gyrus and posterior superior temporal sulcus. Frontiers in human neuroscience. 2010;4:176 Epub 2010/12/15. 10.3389/fnhum.2010.00176 21151362 PMC2995581

[64] Habas C, Guillevin R, Abanou A. Functional connectivity of the superior human temporal sulcus in the brain resting state at 3T. Neuroradiology. 2011;53(2):129–40. Epub 2010/10/07. 10.1007/s00234-010-0775-5 20924756

[65] Gschwind M, Pourtois G, Schwartz S, Van De Ville D, Vuilleumier P. White-matter connectivity between face-responsive regions in the human brain. Cerebral cortex. 2012;22(7):1564–76. Epub 2011/09/07. 10.1093/cercor/bhr226 21893680

[66] Martin A, Santos LR. The origins of belief representation: monkeys fail to automatically represent others' beliefs. Cognition. 2014;130(3):300–8. Epub 2014/01/01. 10.1016/j.cognition.2013.11.016 24374209 PMC3963482

[67] Roland PE. The posterior parietal association cortex in man. Behav Brain Sci. 1980;3(4):513–4. 10.1017/s0140525x00006488

[68] Wright RD, Ward LM. Orienting of attention. Oxford, UK: Oxford University Press; 2008.

[69] Milner D, Goodale M. The visual brain in action. 2nd ed D'Esposito M, Schacter DL, Driver J, Treisman A, Robbins T, Weiskrantz L, editors. Oxford, UK: Oxford University Press; 2006.

[70] Frith CD, Frith U. The neural basis of mentalizing. Neuron. 2006;50(4):531–4. 10.1016/j.neuron.2006.05.001 . 16701204

[71] Mar RA. The neural bases of social cognition and story comprehension. Annual review of psychology. 2011;62:103–34. Epub 2010/12/04. 10.1146/annurev-psych-120709-145406 21126178

[72] Bruce C, Desimone R, Gross CG. Visual properties of neurons in a polysensory area in superior temporal sulcus of the macaque. Journal of neurophysiology. 1981;46(2):369–84. Epub 1981/08/01. 6267219. 6267219 10.1152/jn.1981.46.2.369

[73] Oram MW, Perrett DI. Responses of anterior superior temporal polysensory (STPa) neurons to "biological motion" stimuli. Journal of cognitive neuroscience. 1994;6(2):99–116. Epub 1994/04/01. 10.1162/jocn.1994.6.2.99 23962364

[74] Kilintari M, Raos V, Savaki HE. Involvement of the superior temporal cortex in action execution and action observation. The Journal of neuroscience: the official journal of the Society for Neuroscience. 2014;34(27):8999–9011. Epub 2014/07/06. 10.1523/JNEUROSCI.0736-14.2014 24990920 PMC6608254

[75] Luh KE, Butter CM, Buchtel HA. Impairments in orienting to visual stimuli in monkeys following unilateral lesions of the superior sulcal polysensory cortex. Neuropsychologia. 1986;24(4):461–70. Epub 1986/01/01. 10.1016/0028-3932(86)90091-6 . 3774132

[76] Hikosaka K, Iwai E, Saito H, Tanaka K. Polysensory properties of neurons in the anterior bank of the caudal superior temporal sulcus of the macaque monkey. Journal of neurophysiology. 1988;60(5):1615–37. Epub 1988/11/01. 2462027. 2462027 10.1152/jn.1988.60.5.1615

[77] Noonan MP, Sallet J, Mars RB, Neubert FX, O'Reilly JX, Andersson JL, et al A neural circuit covarying with social hierarchy in macaques. PLoS biology. 2014;12(9):e1001940 10.1371/journal.pbio.1001940 25180883 PMC4151964

[78] Yoshida K, Saito N, Iriki A, Isoda M. Representation of others' action by neurons in monkey medial frontal cortex. Current biology: CB. 2011;21(3):249–53. Epub 2011/01/25. 10.1016/j.cub.2011.01.004 21256015

[79] Yoshida K, Saito N, Iriki A, Isoda M. Social error monitoring in macaque frontal cortex. Nature neuroscience. 2012;15(9):1307–12. Epub 2012/08/07. 10.1038/nn.3180 22864610

[80] Schilbach L, Wilms M, Eickhoff SB, Romanzetti S, Tepest R, Bente G, et al Minds made for sharing: initiating joint attention recruits reward-related neurocircuitry. Journal of cognitive neuroscience. 2010;22(12):2702–15. Epub 2009/11/26. 10.1162/jocn.2009.21401 19929761

[81] Iacoboni M, Lieberman MD, Knowlton BJ, Molnar-Szakacs I, Moritz M, Throop CJ, et al Watching social interactions produces dorsomedial prefrontal and medial parietal BOLD fMRI signal increases compared to a resting baseline. NeuroImage. 2004;21(3):1167–73. Epub 2004/03/10. 10.1016/j.neuroimage.2003.11.013 15006683

[82] Schnell K, Bluschke S, Konradt B, Walter H. Functional relations of empathy and mentalizing: an fMRI study on the neural basis of cognitive empathy. NeuroImage. 2011;54(2):1743–54. Epub 2010/08/24. 10.1016/j.neuroimage.2010.08.024 20728556

[83] Saxe R, Powell LJ. It's the thought that counts: specific brain regions for one component of theory of mind. Psychological science. 2006;17(8):692–9. Epub 2006/08/18. 10.1111/j.1467-9280.2006.01768.x 16913952

[84] Evangeliou MN, Raos V, Galletti C, Savaki HE. Functional imaging of the parietal cortex during action execution and observation. Cerebral cortex. 2009;19(3):624–39. Epub 2008/07/22. 10.1093/cercor/bhn116 . 18641087

[85] Horn A, Ostwald D, Reisert M, Blankenburg F. The structural-functional connectome and the default mode network of the human brain. NeuroImage. 2014;102 Pt 1:142–51. Epub 2013/10/09. 10.1016/j.neuroimage.2013.09.069 24099851

[86] Kobayashi Y, Amaral DG. Macaque monkey retrosplenial cortex: II. Cortical afferents. The Journal of comparative neurology. 2003;466(1):48–79. 10.1002/cne.10883 14515240

[87] Parvizi J, Van Hoesen GW, Buckwalter J, Damasio A. Neural connections of the posteromedial cortex in the macaque. Proceedings of the National Academy of Sciences of the United States of America. 2006;103(5):1563–8. 10.1073/pnas.0507729103 16432221 PMC1345704

[88] Wegener D, Freiwald WA, Kreiter AK. The influence of sustained selective attention on stimulus selectivity in macaque visual area MT. The Journal of neuroscience: the official journal of the Society for Neuroscience. 2004;24(27):6106–14. Epub 2004/07/09. 10.1523/JNEUROSCI.1459-04.2004 15240802 PMC6729659

[89] Jezzard P, Balaban RS. Correction for geometric distortion in echo planar images from B0 field variations. Magnetic resonance in medicine: official journal of the Society of Magnetic Resonance in Medicine / Society of Magnetic Resonance in Medicine. 1995;34(1):65–73. 10.1002/mrm.1910340111 7674900

[90] Masamoto K, Kanno I. Anesthesia and the quantitative evaluation of neurovascular coupling. Journal of cerebral blood flow and metabolism: official journal of the International Society of Cerebral Blood Flow and Metabolism. 2012;32(7):1233–47. Epub 2012/04/19. 10.1038/jcbfm.2012.50 22510601 PMC3390804

[91] Deshpande G, Kerssens C, Sebel PS, Hu X. Altered local coherence in the default mode network due to sevoflurane anesthesia. Brain research. 2010;1318:110–21. Epub 2010/01/12. 10.1016/j.brainres.2009.12.075 20059988 PMC2845285

[92] Martuzzi R, Ramani R, Qiu M, Rajeevan N, Constable RT. Functional connectivity and alterations in baseline brain state in humans. NeuroImage. 2010;49(1):823–34. Epub 2009/07/28. 10.1016/j.neuroimage.2009.07.028 19631277 PMC2764802

[93] Hutchison RM, Hutchison M, Manning KY, Menon RS, Everling S. Isoflurane induces dose-dependent alterations in the cortical connectivity profiles and dynamic properties of the brain's functional architecture. Human brain mapping. 2014;35(12):5754–75. Epub 2014/07/22. 10.1002/hbm.22583 25044934 PMC6869297

[94] Hutchison RM, Gallivan JP, Culham JC, Gati JS, Menon RS, Everling S. Functional connectivity of the frontal eye fields in humans and macaque monkeys investigated with resting-state fMRI. Journal of neurophysiology. 2012;107(9):2463–74. Epub 2012/02/03. 10.1152/jn.00891.2011 22298826

[95] Iglewicz B, Hoaglin DC. How to detect and handle outliers. Mykytka EF, editor. Milwaukee, WI: ASQC Quality Press; 1993.

[96] Dale AM, Fischl B, Sereno MI. Cortical surface-based analysis. I. Segmentation and surface reconstruction. NeuroImage. 1999;9(2):179–94. Epub 1999/02/05. 10.1006/nimg.1998.0395 9931268

[97] Fischl B, Sereno MI, Dale AM. Cortical surface-based analysis. II: Inflation, flattening, and a surface-based coordinate system. NeuroImage. 1999;9(2):195–207. Epub 1999/02/05. 10.1006/nimg.1998.0396 9931269

[98] Teichert T, Grinband J, Hirsch J, Ferrera VP. Effects of heartbeat and respiration on macaque fMRI: implications for functional connectivity. Neuropsychologia. 2010;48(7):1886–94. Epub 2009/12/09. 10.1016/j.neuropsychologia.2009.11.026 19969009 PMC2876227

[99] Glover GH, Li TQ, Ress D. Image-based method for retrospective correction of physiological motion effects in fMRI: RETROICOR. Magnetic resonance in medicine: official journal of the Society of Magnetic Resonance in Medicine / Society of Magnetic Resonance in Medicine. 2000;44(1):162–7. Epub 2000/07/14. 10.1002/1522-2594(200007)44:1<162::aid-mrm23>3.3.co;2-5 10893535

[100] Verstynen TD, Deshpande V. Using pulse oximetry to account for high and low frequency physiological artifacts in the BOLD signal. NeuroImage. 2011;55(4):1633–44. Epub 2011/01/13. 10.1016/j.neuroimage.2010.11.090 21224001

[101] Silver NC, Dunlap WP. Averaging correlation coefficients: should Fisher's z transformation be used? J Appl Psychol. 1987;72(1):146–8. 10.1037/0021-9010.72.1.146

[102] Hodges JL Jr., Lehmann EL. Estimates of location based on rank tests. Ann Math Stat. 1963;34(2):598–611. 10.1214/aoms/1177704172

[103] Hagler DJ Jr., Saygin AP, Sereno MI. Smoothing and cluster thresholding for cortical surface-based group analysis of fMRI data. NeuroImage. 2006;33(4):1093–103. Epub 2006/10/03. 10.1016/j.neuroimage.2006.07.036 17011792 PMC1785301

[104] Nichols T, Brett M, Andersson J, Wager T, Poline JB. Valid conjunction inference with the minimum statistic. NeuroImage. 2005;25(3):653–60. Epub 2005/04/06. 10.1016/j.neuroimage.2004.12.005 15808966

[105] Popivanov ID, Jastorff J, Vanduffel W, Vogels R. Stimulus representations in body-selective regions of the macaque cortex assessed with event-related fMRI. NeuroImage. 2012;63(2):723–41. Epub 2012/07/17. 10.1016/j.neuroimage.2012.07.013 22796995

[106] Golub GH, van Loan CF. Matrix computations. 3 ed Baltimore MD: Johns Hopkins University Press; 1996.

[107] Salvador R, Suckling J, Coleman MR, Pickard JD, Menon D, Bullmore E. Neurophysiological architecture of functional magnetic resonance images of human brain. Cerebral cortex. 2005;15(9):1332–42. Epub 2005/01/07. 10.1093/cercor/bhi016 15635061

[108] Bellec P, Perlbarg V, Jbabdi S, Pelegrini-Issac M, Anton JL, Doyon J, et al Identification of large-scale networks in the brain using fMRI. NeuroImage. 2006;29(4):1231–43. Epub 2005/10/26. 10.1016/j.neuroimage.2005.08.044 16246590

[109] Supekar K, Musen M, Menon V. Development of large-scale functional brain networks in children. PLoS biology. 2009;7(7):e1000157 Epub 2009/07/22. 10.1371/journal.pbio.1000157 19621066 PMC2705656

[110] Benjamini Y, Hochberg Y. Controlling the False Discovery Rate: a practical and powerful approach to multiple testing. J Roy Stat Soc B. 1995;57(1):289–300. 10.2307/2346101

[111] Jaccard P. The distribution of the flora in the alpine zone. New Phytologist. 1912;11(2):37–50. 10.1111/j.1469-8137.1912.tb05611.x

[112] Fischl B, Sereno MI, Tootell RB, Dale AM. High-resolution intersubject averaging and a coordinate system for the cortical surface. Human brain mapping. 1999;8(4):272–84. Epub 2000/01/05. 10.1002/(SICI)1097-0193(1999)8:4<272::AID-HBM10>3.0.CO;2-4 10619420 PMC6873338

[113] van Essen DC. Windows on the brain: the emerging role of atlases and databases in neuroscience. Current opinion in neurobiology. 2002;12(5):574–9. Epub 2002/10/09. 10.1016/s0959-4388(02)00361-6 12367638

[114] van Essen DC, Glasser MF, Dierker DL, Harwell J. Cortical parcellations of the macaque monkey analyzed on surface-based atlases. Cerebral cortex. 2012;22(10):2227–40. Epub 2011/11/05. 10.1093/cercor/bhr290 22052704 PMC3500860

[115] Frey S, Pandya DN, Chakravarty MM, Bailey L, Petrides M, Collins DL. An MRI based average macaque monkey stereotaxic atlas and space (MNI monkey space). NeuroImage. 2011;55(4):1435–42. Epub 2011/01/25. 10.1016/j.neuroimage.2011.01.040 21256229

