# Supplementary figures and images for: Face Patch Resting State Networks Link Face Processing to Social Cognition
Source: PLoS Biol. 2015 Sep 8;13(9):e1002245. doi: 10.1371/journal.pbio.1002245 (PMC4562659; doi:10.1371/journal.pbio.1002245)

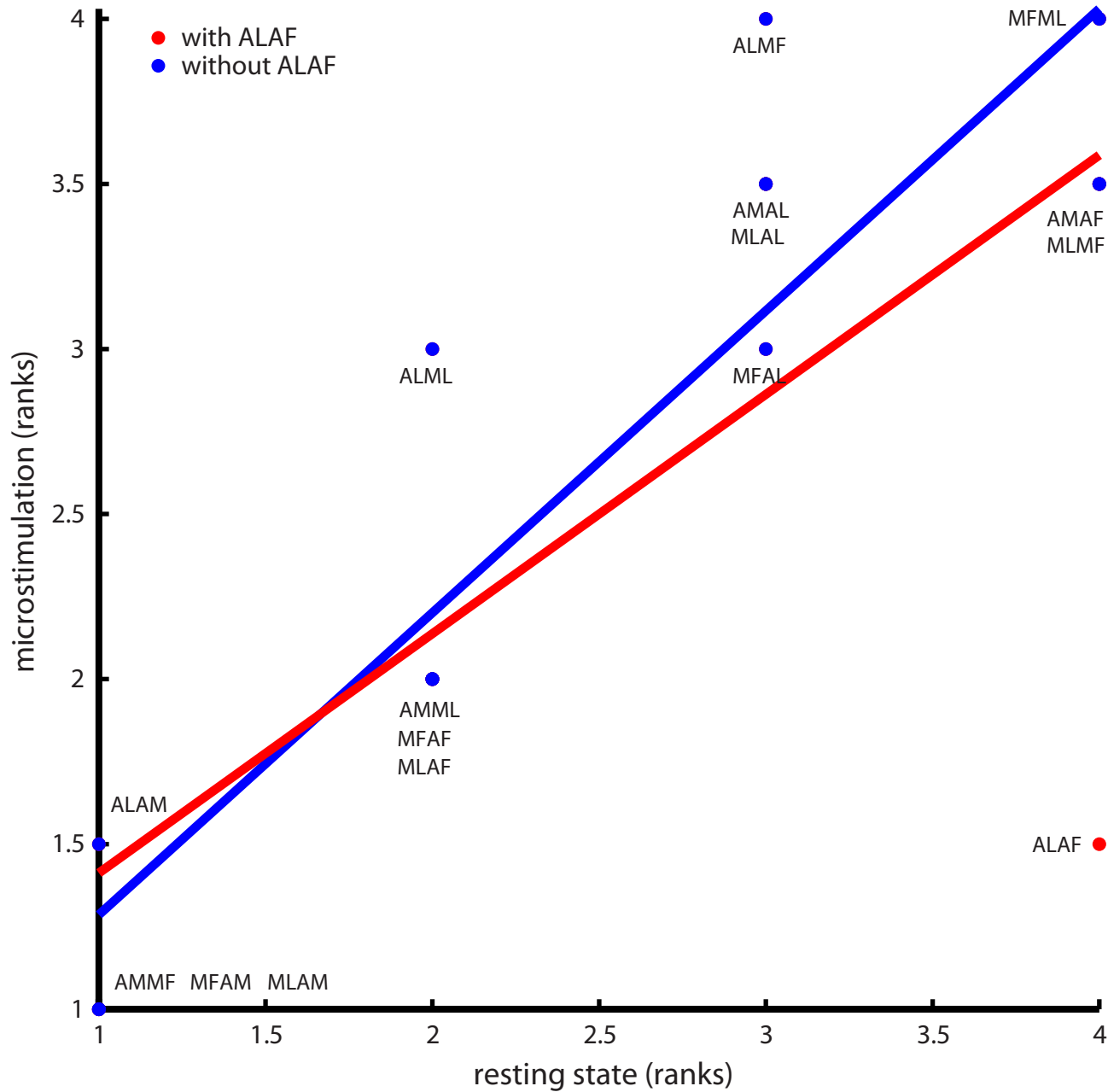

Supplement: S1 Fig — Spearman rank correlations revealed a strong correlation (r = 0.5256, p = 0.0365, red) between the connection strengths obtained through noninvasive rsfMRI and electrical microstimulation [3]. After the exclusion of one extreme data point (AL–AF), the rank correlation rose to r = 0.6314, p = 0.0116 (blue). The same results were obtained using the robust correlation method Shepherd’s Pi (r = 0.6352, p = 0.0219) [33]. To bring rsfMRI and microstimulation connectivity onto the same scale, we first computed the median connectivity strength across animals per connection for the microstimulation data and then computed tied ranks within the columns of this and the resting state connectivity matrices. (PDF) [file pbio.1002245.s001.pdf]

PL

prefrontal  
premotor-parietal  
occipito-temporal

$10^{-20}$   $10^{-50}$   
 $p$

n=4

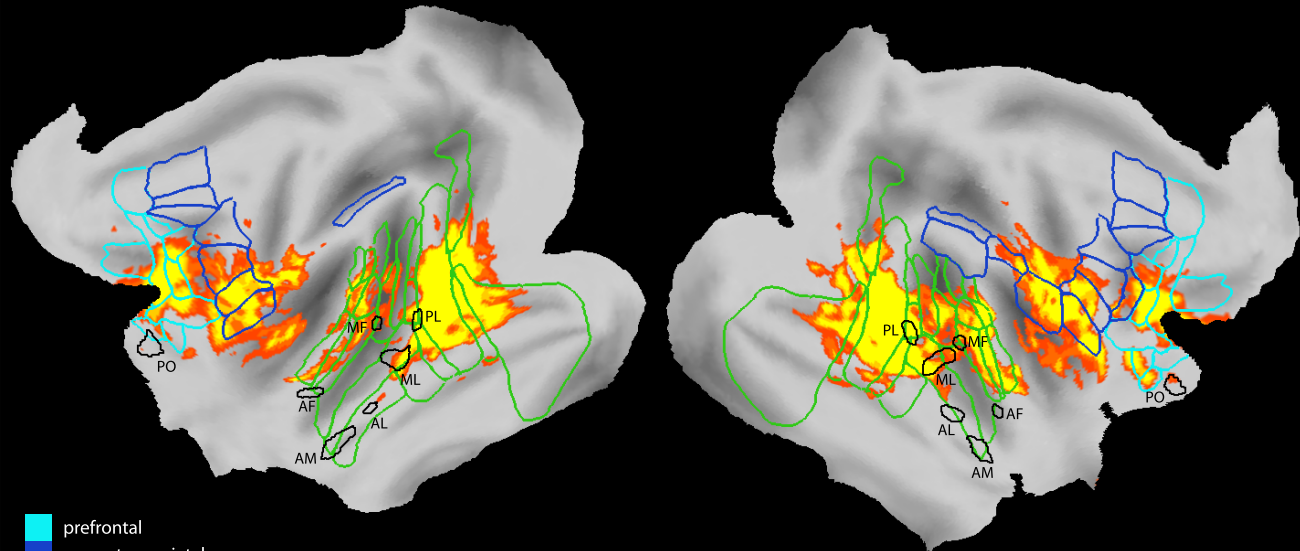

Supplement: S2 Fig — PL, which could be identified in four out of six animals in the study, showed a connectivity pattern (multiple comparisons corrected using cluster size thresholding at p < 0.05) that was similar to that of the other face patches, including connectivity to prefrontal, premotor, and occipitotemporal areas. Results are shown on inflated and flattened left and right hemispheres in F99 space. Representative locations of the face patches are outlined in black. For comparison, three broad networks of connectivity from the main conjunction analysis (including AF, AL, MF, ML, and PO) are highlighted: areas in prefrontal cortex (light blue), a premotor-parietal network (blue), and an occipitotemporal network including ventral stream areas. Areal boundaries are from Lewis & van Essen [35]. Data shown here are publicly available at the Dryad Digital Repository [39]. (PDF) [file pbio.1002245.s002.pdf]

MF

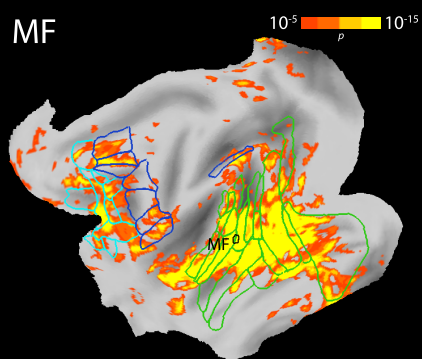

ML

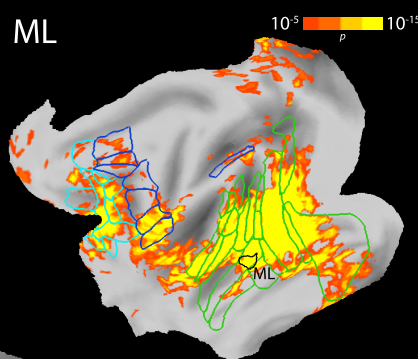

AM

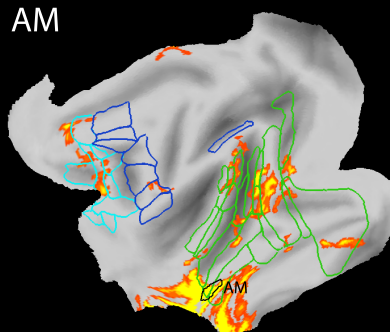

PO

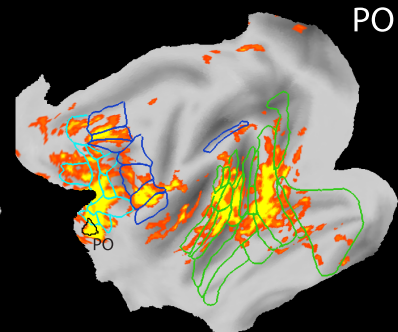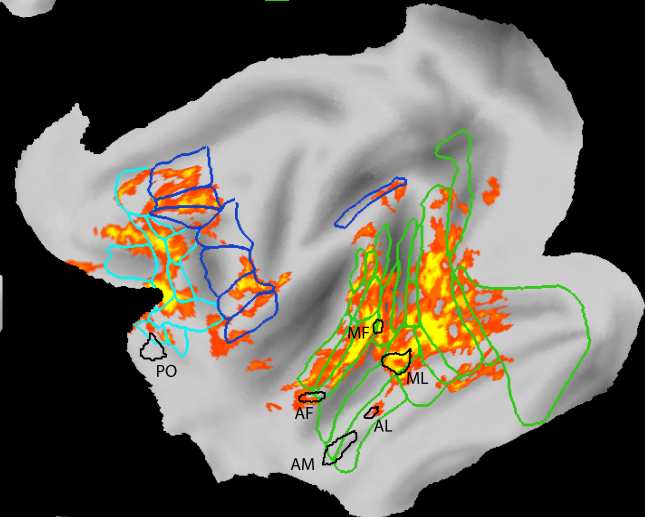

AF

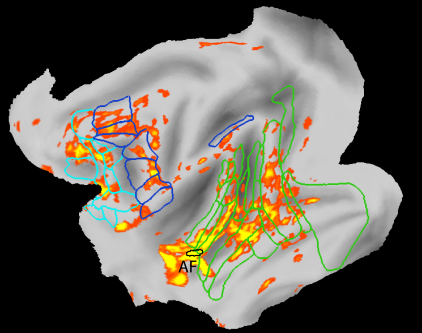

AL

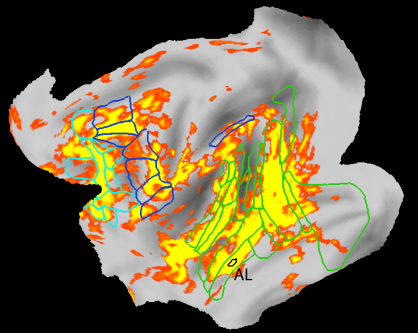

Supplement: S3 Fig — The central panel shows the results of a conjunction analysis of the maps from AF, AL, MF, ML, and PO (multiple comparisons corrected using cluster size thresholding at p < 0.05) on an inflated and flattened left hemisphere in F99 space. Highlighted are three broad networks of connectivity: areas in prefrontal cortex (light blue), a premotor-parietal network (blue), and an occipitotemporal network including ventral stream areas (green). The surrounding panels show connectivity maps of the individual face patches (multiple comparisons corrected using cluster size thresholding at p < 0.05), along with the areal boundaries of the conjunction analysis (light blue, blue, green). Representative locations of the face patches are outlined in black. Areal boundaries are from Lewis & van Essen [35]. See S1 Table for a list of area names. Data shown here are publicly available at the Dryad Digital Repository [39]. (PDF) [file pbio.1002245.s003.pdf]

■ prefrontal  
■ premotor-parietal  
■ occipito-temporal

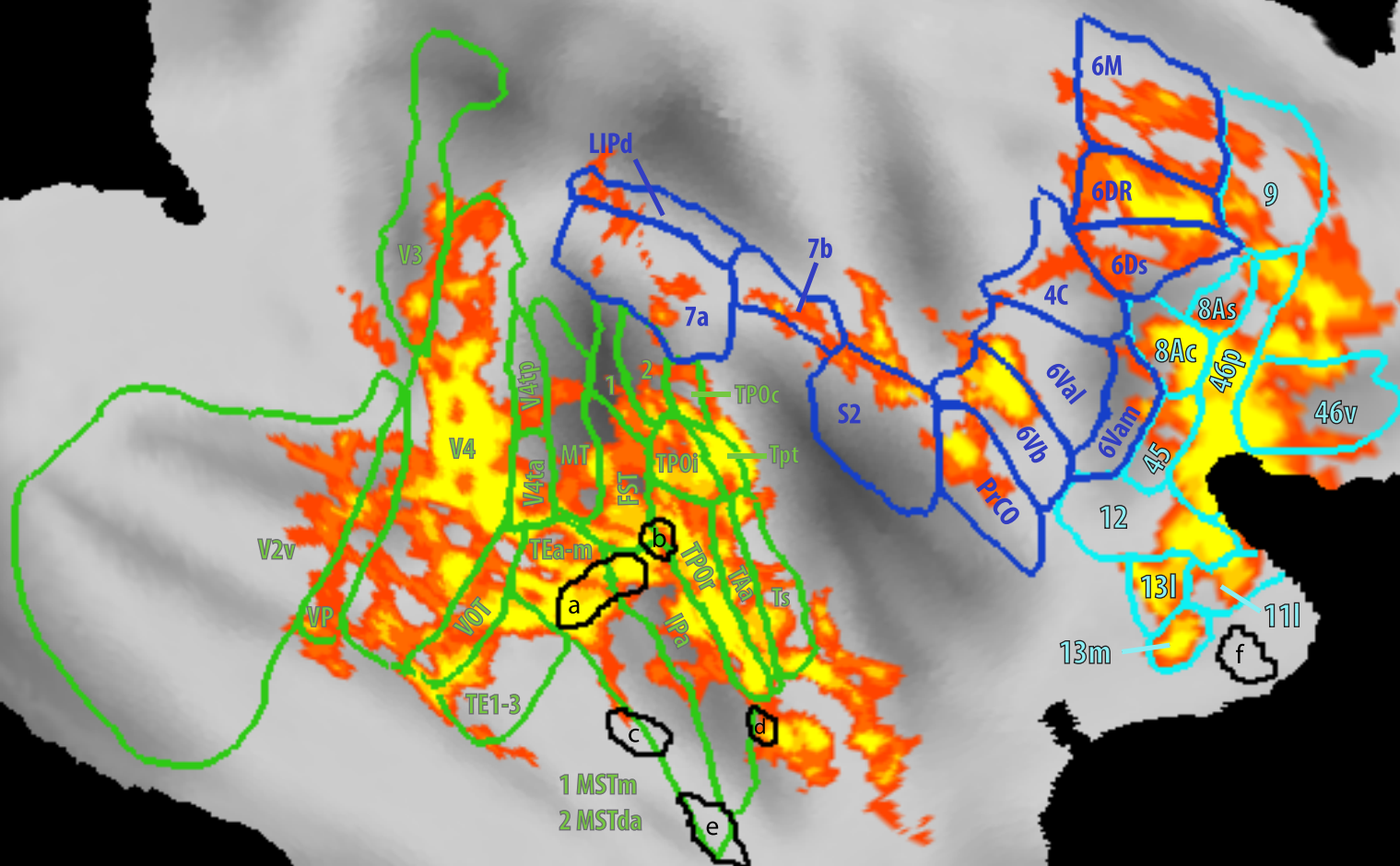

a: ML  
 b: MF  
 c: AL  
 d: AF  
 e: AM  
 f: PO

conjunction  
 $10^{-3}$  ■ ■  $10^{-10}$   
 p

Supplement: S4 Fig — Shown are the results of a conjunction analysis of the maps from AF, AL, MF, ML, and PO (multiple comparisons corrected using cluster size thresholding at p < 0.05) on an inflated and flattened right hemisphere in F99 space. Representative locations of the face patches are outlined in black. Data shown here are publicly available at the Dryad Digital Repository [39]. (PDF) [file pbio.1002245.s004.pdf]

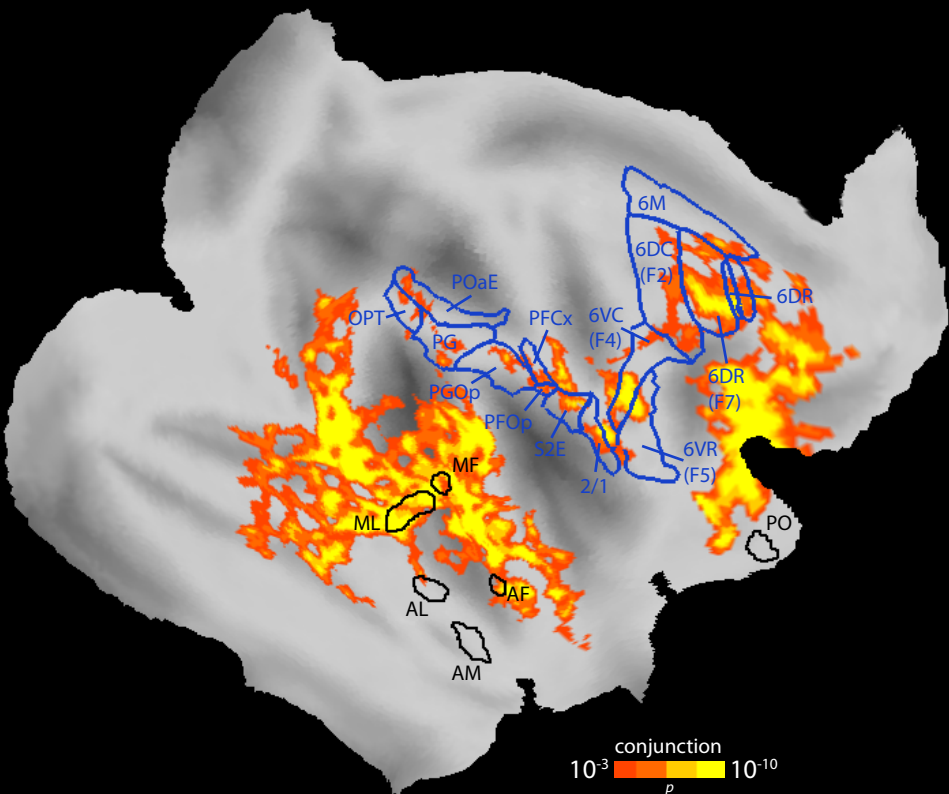

Supplement: S5 Fig — Shown are the results of a conjunction analysis of the maps from AF, AL, MF, ML, and PO (multiple comparisons corrected using cluster size thresholding at p < 0.05) on an inflated and flattened left hemisphere in F99 space. Representative locations of the face patches are outlined in black. Data shown here are publicly available at the Dryad Digital Repository [39]. (PDF) [file pbio.1002245.s005.pdf]

a: ML  
b: MF  
c: AL  
d: AF  
e: AM  
f: PO

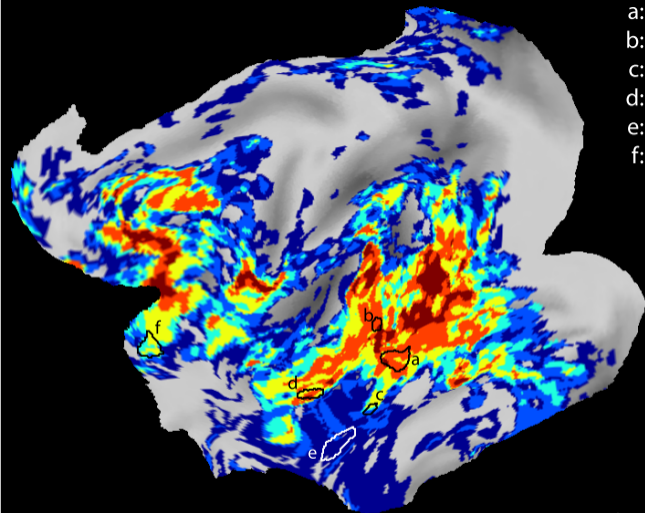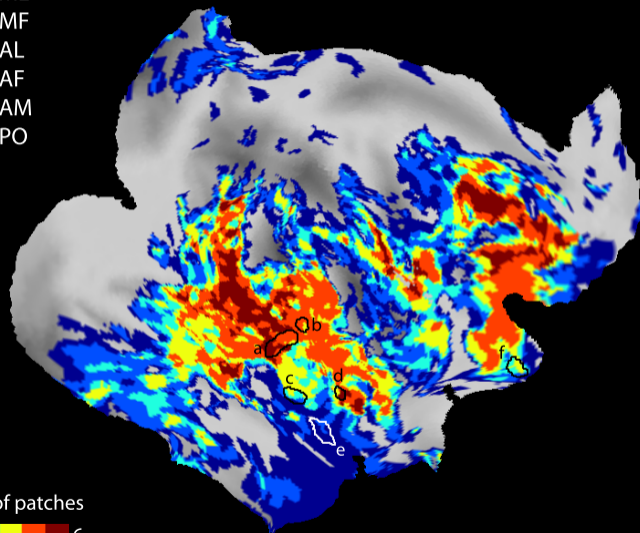

number of patches

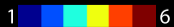

Supplement: S6 Fig — For each vertex, we quantified how many face patches were functionally connected after correction for multiple comparisons using cluster size thresholding at p < 0.05 (cf. outer panels of Fig 2 & S3 Fig). Results are shown on inflated and flattened left and right hemispheres in F99 space. Representative locations of the face patches are outlined in black/white. Data shown here are publicly available at the Dryad Digital Repository [39]. (PDF) [file pbio.1002245.s006.pdf]

prefrontal  
premotor-parietal  
occipito-temporal

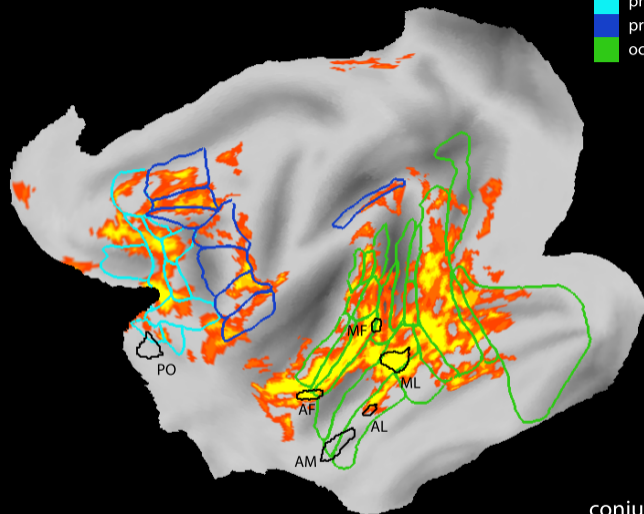

conjunction  
 $10^{-3}$   $10^{-10}$   
 $p$

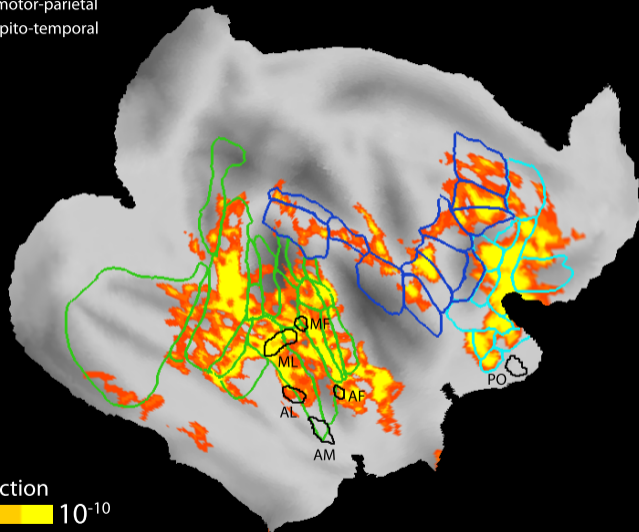

Supplement: S7 Fig — Shown are the results of a conjunction analysis of the maps from AF, AL, MF, and ML (multiple comparisons corrected using cluster size thresholding at p < 0.05) on inflated and flattened left and right hemispheres in F99 space. Highlighted are three broad networks of connectivity from the main conjunction analysis including the orbitofrontal face patch PO: areas in prefrontal cortex (light blue), a premotor-parietal network (blue), and an occipitotemporal network including ventral stream areas (green). Areal boundaries are from Lewis & van Essen [35]. Representative locations of the face patches are outlined in black. Data shown here are publicly available at the Dryad Digital Repository [39]. (PDF) [file pbio.1002245.s007.pdf]

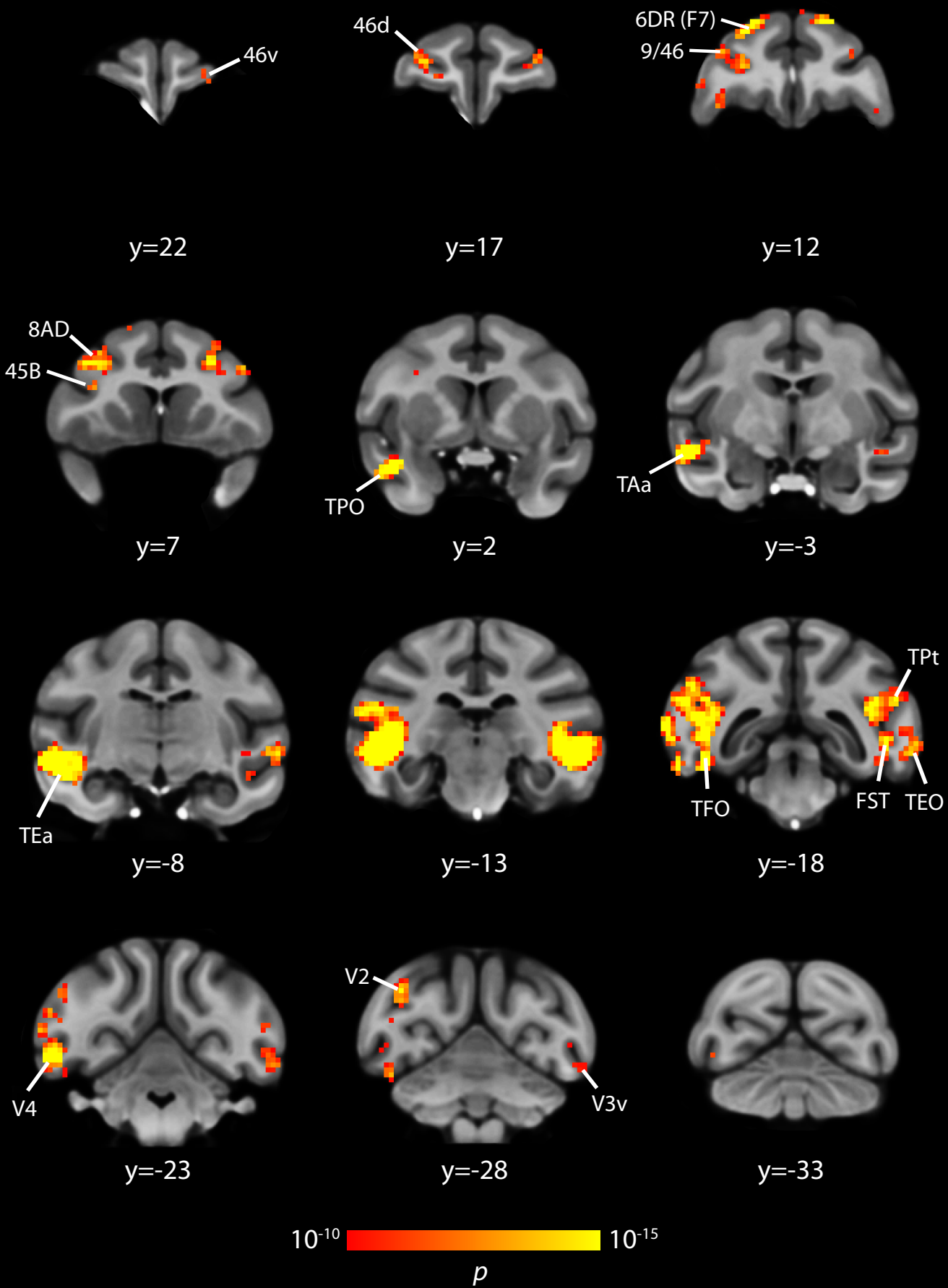

Supplement: S8 Fig — Shown are the results of a conjunction analysis (uncorrected) of the rsfMRI maps of bilateral face patches AF, AL, MF, ML, and PO, overlaid on coronal slices of the MNI-Paxinos template brain, in radiological convention (left is right). Coordinates are relative to the center of the anterior commissure. Area labels are based on Paxinos et al. [38]. (PDF) [file pbio.1002245.s008.pdf]

a) AL, ML, PO > AF, MF

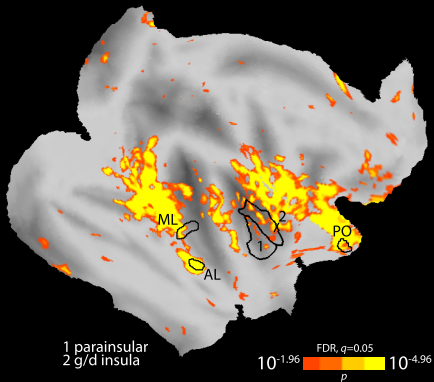

b) AF, AL > MF, ML, PO

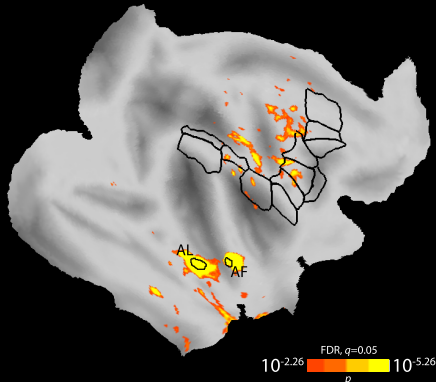

c) AM > AF, AL, MF, ML, PO

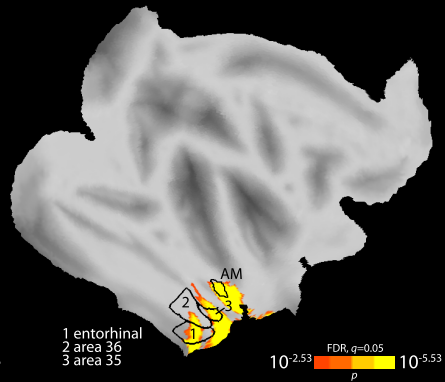

Supplement: S9 Fig — (a) connectivity to the insula was more prominent for AL, ML, and PO than for MF and AF; (b) AL and AF connectivity extended more posteriorly on the dorsolateral surface towards the central sulcus than any of the other face patches (the premotor-parietal network from the main conjunction analysis is shown for reference); (c) only AM showed connectivity to medial temporal lobe structures (entorhinal cortex, perirhinal cortical areas 35 and 36). All contrasts were corrected for multiple comparisons using a FDR at q = 0.05. Results are shown on an inflated and flattened right hemisphere in F99 space, negative differences are truncated for display purposes. Representative locations of the face patches are outlined in black. Areal boundaries are from Lewis & van Essen [35]. Data shown here are publicly available at the Dryad Digital Repository [39]. (PDF) [file pbio.1002245.s009.pdf]

## a) posterior STS

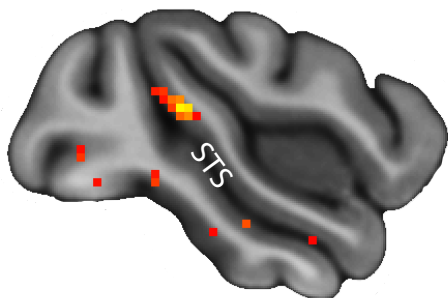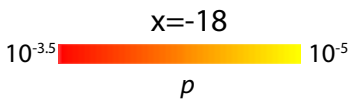

## b) dmPFC

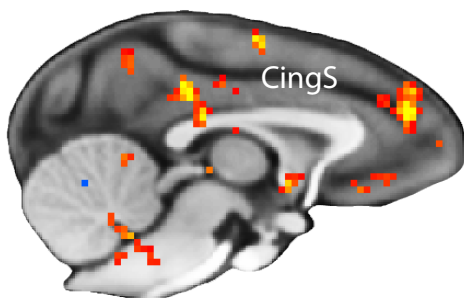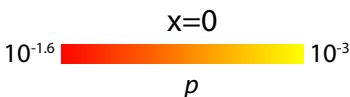

Supplement: S10 Fig — (a) Voxels in area TPO in the dorsal bank of the posterior STS that show significant connectivity both with the PPC and AM at p < 10−3.5, uncorrected. (c) Voxels in dmPFC and medial PPC that show significant connectivity both with the PPC and AM at p < 10−1.6, uncorrected. The overlap between the resting state network of AM and the DMN was highly consistent with that of the other face patches, localizing to three areas known to support high-level social cognition in humans. Results are overlaid on the MNI-Paxinos template brain. Coordinates are relative to the center of the anterior commissure. (PDF) [file pbio.1002245.s010.pdf]

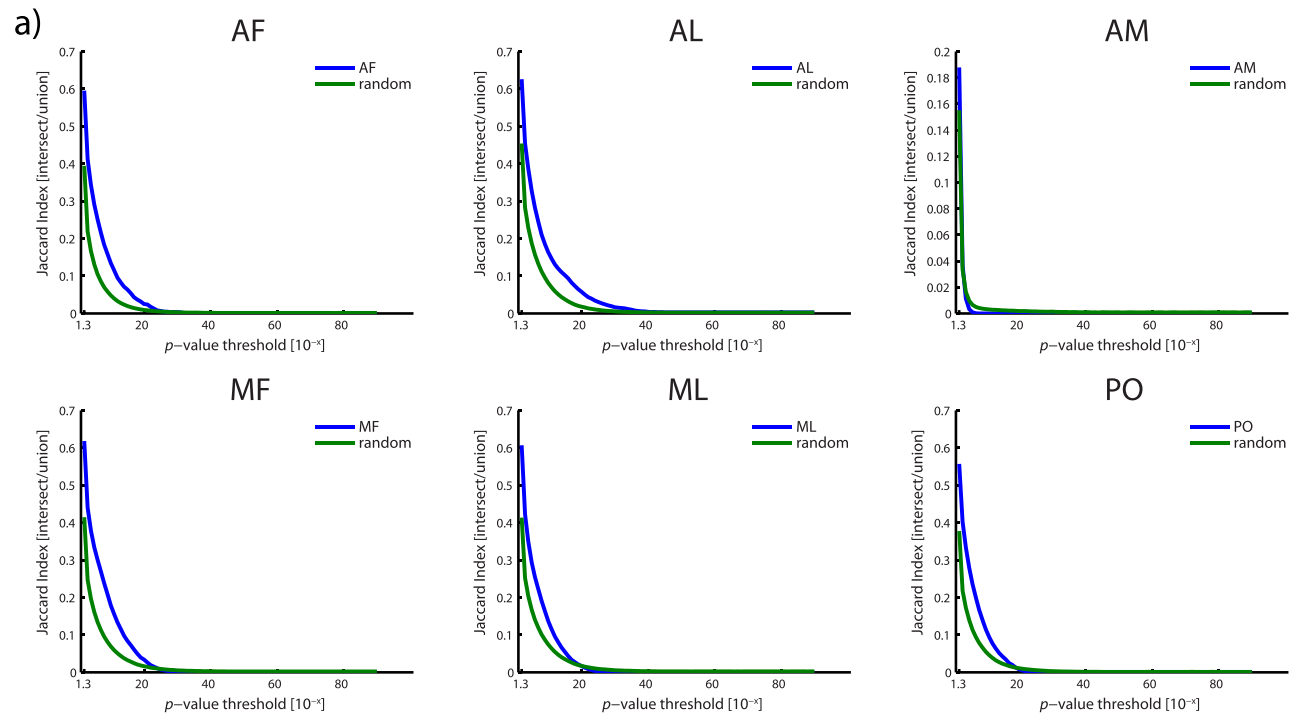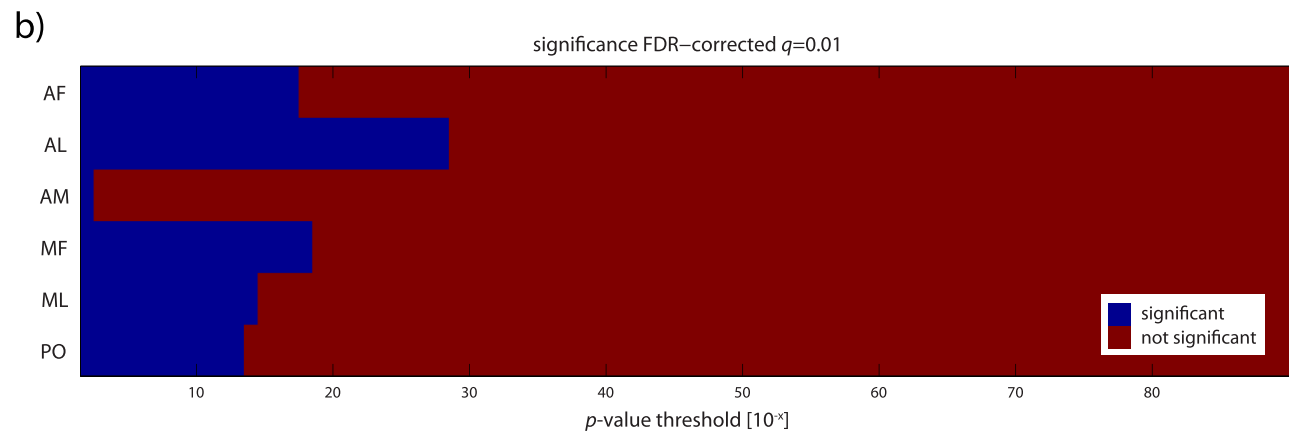

Supplement: S11 Fig — a) Results of the permutation tests for significant overlap between the DMN and individual FPRSNs, quantified as Jaccard Indices, over a wide range of statistical thresholds. The blue lines show the empirically observed overlap, while the green lines show the average degree of overlap between the respective face patch map and 5,000 randomly generated noise maps with spatial smoothness and number of significant voxels matched to those of the DMN map at each threshold. b) After correction for multiple comparisons (FDR, q = 0.01), there was significant overlap between the DMN and each of the FPRSNs until thresholds were so conservative that the likelihood of overlap was minimized. (PDF) [file pbio.1002245.s011.pdf]

# faces vs. objects overlap with DMN

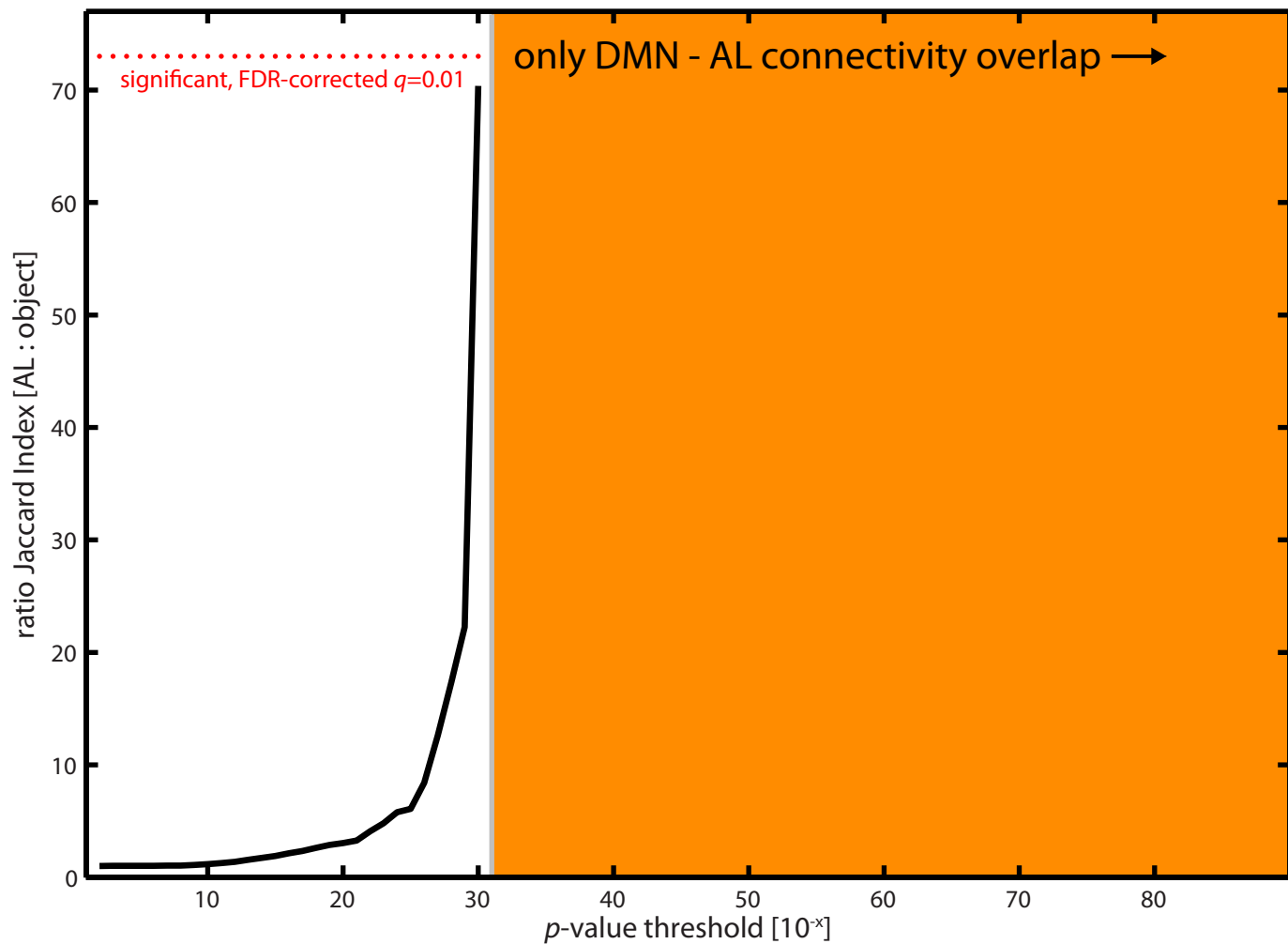

Supplement: S12 Fig — We compared the overlap between the resting state networks of the face patch AL and the DMN to the overlap between the resting state networks of a nearby object patch and the DMN. The ratio of Jaccard Indices for AL-DMN to object patch-DMN rapidly increases as statistical thresholds get more conservative. For most thresholds tested, there was no overlap between the object patch resting state network and the DMN (orange area). For the remaining thresholds, χ 2-tests of the ratio of overlapping to nonoverlapping DMN voxels between AL and the object patch showed that face patch connectivity overlap always exceeded object patch connectivity overlap (corrected for multiple comparisons using a FDR at q = 0.01). (PDF) [file pbio.1002245.s012.pdf]
