# Supplementary material for: Face Patch Resting State Networks Link Face Processing to Social Cognition
Source: PLoS Biol. 2015 Sep 8;13(9):e1002245. doi: 10.1371/journal.pbio.1002245 (PMC4562659; doi:10.1371/journal.pbio.1002245)
Supplement: S1 Table — Area names are from Lewis & van Essen [35]. (DOC) [file pbio.1002245.s013.doc]

| **Prefrontal** | **Premotor-parietal** | **Occipito-temporal** |
| --- | --- | --- |
| 11l | 4C | FST |
| 12 | 6DR | IPa |
| 13l | 6Ds | MSTda |
| 45 | 6M | fMT |
| 46p | 6Val | PA |
| 46v | 6Vb | TAa |
| 8Ac | 7a | TE1-3 |
| 8As | LIPd | TE1-3d |
| 9 | PrCO | TEa-m |
|  |  | TPOc |
|  |  | TPOi |
|  |  | TPOr |
|  |  | Tpt |
|  |  | Ts |
|  |  | V2v |
|  |  | V3 |
|  |  | V4 |
|  |  | V4ta |
|  |  | V4tp |
|  |  | VOT |
|  |  | VP |
